# Self-eating: friend or foe? The emerging role of autophagy in fibrotic diseases

**DOI:** 10.7150/thno.47826

**Published:** 2020-06-29

**Authors:** Yajing Li, Runping Liu, Jianzhi Wu, Xiaojiaoyang Li

**Affiliations:** 1 School of Life Sciences, Beijing University of Chinese Medicine, 11 Bei San Huan Dong Lu, Beijing, 100029, China.; 2 School of Chinese Materia Medica, Beijing University of Chinese Medicine, 11 Bei San Huan Dong Lu, Beijing, 100029, China.

**Keywords:** Fibrosis, autophagy, TGF-β pathway, cell senescence, protein degradation.

## Abstract

Fibrosis occurs in most human organs including the liver, lung, heart and kidney, and is crucial for the progression of most chronic diseases. As an indispensable catabolic process for intracellular quality control and homeostasis, autophagy occurs in most mammalian cells and is implicated in many biological processes including fibrogenesis. Although advances have been made in understanding autophagy process, the potential role of autophagy in fibrotic diseases remains controversial and has recently attracted a great deal of attention. In the current review, we summarize the commonalities of autophagy affecting different types of fibrosis in different organs, including the liver, lung, heart, and kidney as well as in cystic fibrosis, systematically outline the contradictory results and highlight the distinct role of autophagy during the various stages of fibrosis. In summary, the exact role autophagy plays in fibrogenesis depends on specific cell types and different stimuli, and identifying and evaluating the pathogenic contribution of autophagy in fibrogenesis will promote the discovery of novel therapeutic strategies for the clinical management of these fibrotic diseases.

## Fibrosis

Under the influence of exogenous stimuli, such as pathogens and toxins, and endogenous stimuli, including cytokines and certain metabolites, fibrosis gradually occurs in most human organs including the liver, lung, kidney, and heart, and participates in the progression of almost all chronic diseases, which are associated with high morbidity and mortality due to unclear pathogenesis and limited therapeutic options. Following tissue injury, epithelial and endothelial cells release chemokines, cytokines, damage-associated molecular patterns (DAMPs) and growth factors, leading to increased vessel permeability and infiltration of immune cells. In the initial inflammation phase, resident fibroblasts, fibrocytes and epithelial cells undergoing epithelial-mesenchymal transition (EMT) transform into α-smooth muscle actin (α-SMA)-expressing and extracellular matrix (ECM)-producing myofibroblasts. Stimulated myofibroblasts, epithelial and endothelial cells also produce matrix metalloproteinases (MMPs), contributing to the balance between ECM deposition and degradation. In the subsequent remodeling phase, myofibroblasts continuously produce ECM proteins and trigger wound contraction accompanied by angiogenesis 1. The physiological degradation of ECM and re-epithelialization are crucial features of tissue repair and remodeling, while under pathological conditions, persistent myofibroblast activation caused by chronic injury and unresolved inflammation results in the persistent production of ECM as well as tissue inhibitor of metalloproteinases (TIMPs), inhibitors of MMPs 2. The excessive deposition of ECM leads to a progressively irreversible fibrotic healing response, which not only results in the replacement of normal parenchymal tissue with fibrotic tissue but also leads to organ dysfunction and even failure.

## Autophagy

**Figure 1:**
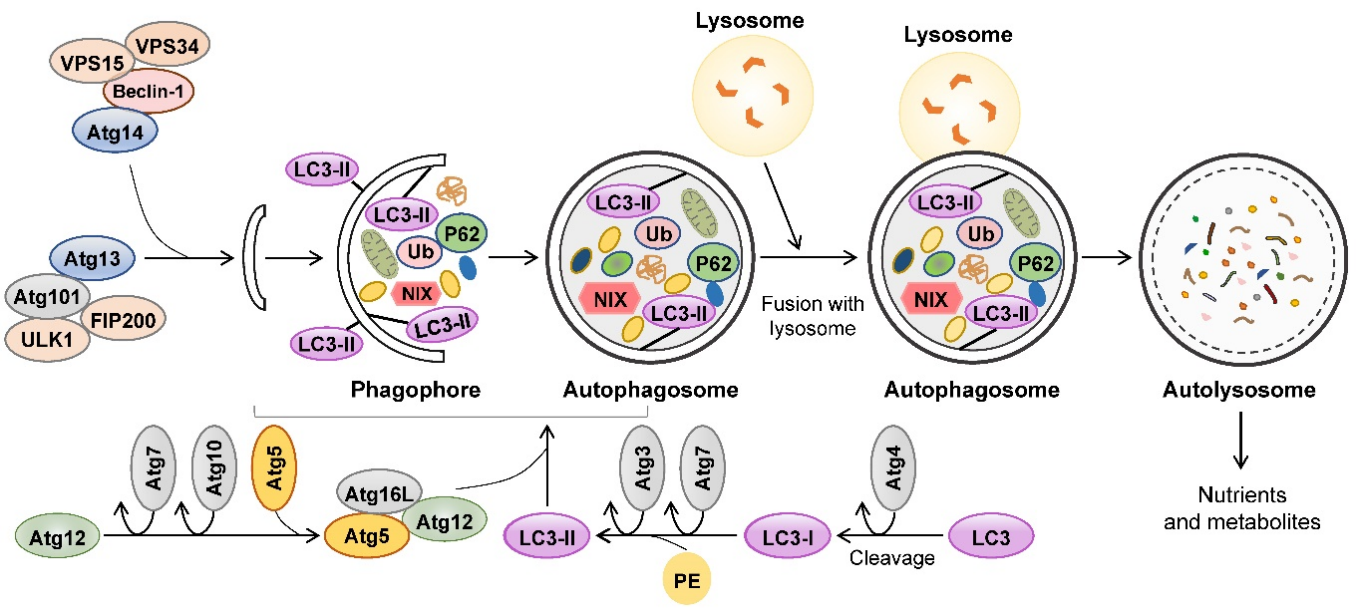
** The feature of autophagy.** Autophagy involves the formation of autophagosomes, their fusion with lysosomes and subsequent degradation of autophagolysosomal contents. The formation of phagophore is depending on the interactions between lipid bilayers and recruited ULK complex (composed of Atg13, Atg101, ULK1 and FIP200) and PI3K complex (composed of Atg14, Beclin-1, VPS34 and VPS15). At the same time, two different conjugation systems, LC3 and the complex of Atg12-Atg5-Atg16L are involved in the assembling of autophagosome. After devouring damaged proteins and organelles, mature autophagosome fuses with lysosome to form autolysosome. Finally, the inner contents are degraded by lysosomal hydrolases and released as nutrients and metabolites. Atg: autophagy-related gene; LC3: Light chain 3.

Autophagy is a self-eating catabolic process involving intracellular degradation and recycling that plays a housekeeping role in the maintenance of cellular homeostasis, especially when cells are undergoing starvation or other stress stimulation 3. Autophagy participates in energy metabolism, regulates cellular growth and differentiation, and is responsible for the clearance of misfolded proteins, damaged organelles and lipid droplets (LDs) 4. Autophagy occurs in most mammalian cells and involves a series of specific steps: autophagosome formation, secretion of cargo, formation and degradation of autophagosome. There are three primary types of autophagy depending on lysosomal functions, including macroautophagy, microautophagy and chaperone-mediated autophagy 5. Generally, autophagy is often referred to as macroautophagy, which majorly eliminates damaged cellular organelles and debris. In the process of macroautophagy, a vesicular structure called an autophagosome is formed after a phagophore, which is a double or multimembrane structure and swallows organelles sequestrated by cytoplasm **(Figure 1)**. Conserved factors called autophagy-related gene (Atg) proteins, including Atg3, Atg5, Atg6, Atg7, Atg9, Atg12, Atg14 and Atg16, are required for autophagosome formation, the first step of autophagy. Light chain 3 (LC3) including LC3A, LC3B and LC3C, as well as Beclin-1, autophagy substrate p62 (p62/SQSTM1) and cathepsin B (CTSB) also play significant roles in autophagosome formation 6. Upon stimulation, LC3 binds to Atg4 and cleavages into LC3-I, which subsequently binds to Atg4 and Atg7 and is cleaved to LC3-II. Subsequently, LC3-II, together with Atg5, Atg7, Atg10, Atg16 and Atg12, as well as Beclin-1 accelerate the formation of the phagophore. Later, p62 functions as a selective autophagy adaptor by simultaneously capturing ubiquitinated cargo and interacting with LC3-II, after which the LC3-II-p62-cargo complex is anchored to the autophagosomal membrane 7. Autophagy flux is a dynamic process involving the formation and clearance of autophagosomes, the phagocytosis of cargo and the formation and degradation of autolysosomes. The increased ratios of LC3-II/LC3-I and LC3-II/p62 are believed to the hallmarks of autophagy flux 8. After devouring damaged proteins and organelles, mature autophagosomes are transited to fuse with the lysosomes and form a single membrane autolysosome mediated by microtubules. Autolysosomes further degrade cargos and release nutrients and metabolites to the cytoplasm for cellular replenishment.

Although advances have been achieved in elucidating the role of autophagy in cell signaling, the clearance of misfolded proteins, lipid metabolism and inflammation progression, the importance of the contradictory roles of autophagy-mediated processes in fibrogenesis in different organs have attracted a great deal of attention. However, there is a dispute regarding how the activation or inhibition of autophagy is involved in the progression of fibrotic diseases and whether tissue/cell-specific autophagy-related genes can be therapeutic targets for the treatment of fibrosis. In this review, we summarize the latest findings with regards to the correlation between autophagy and fibrosis in different main organs, including the liver, lung, heart, kidney as well as cystic fibrosis, and highlight the existing evidence supporting autophagy as a potential target for the treatment of chronic multiorgan fibrotic diseases.

## Liver fibrosis and autophagy

**Figure 2:**
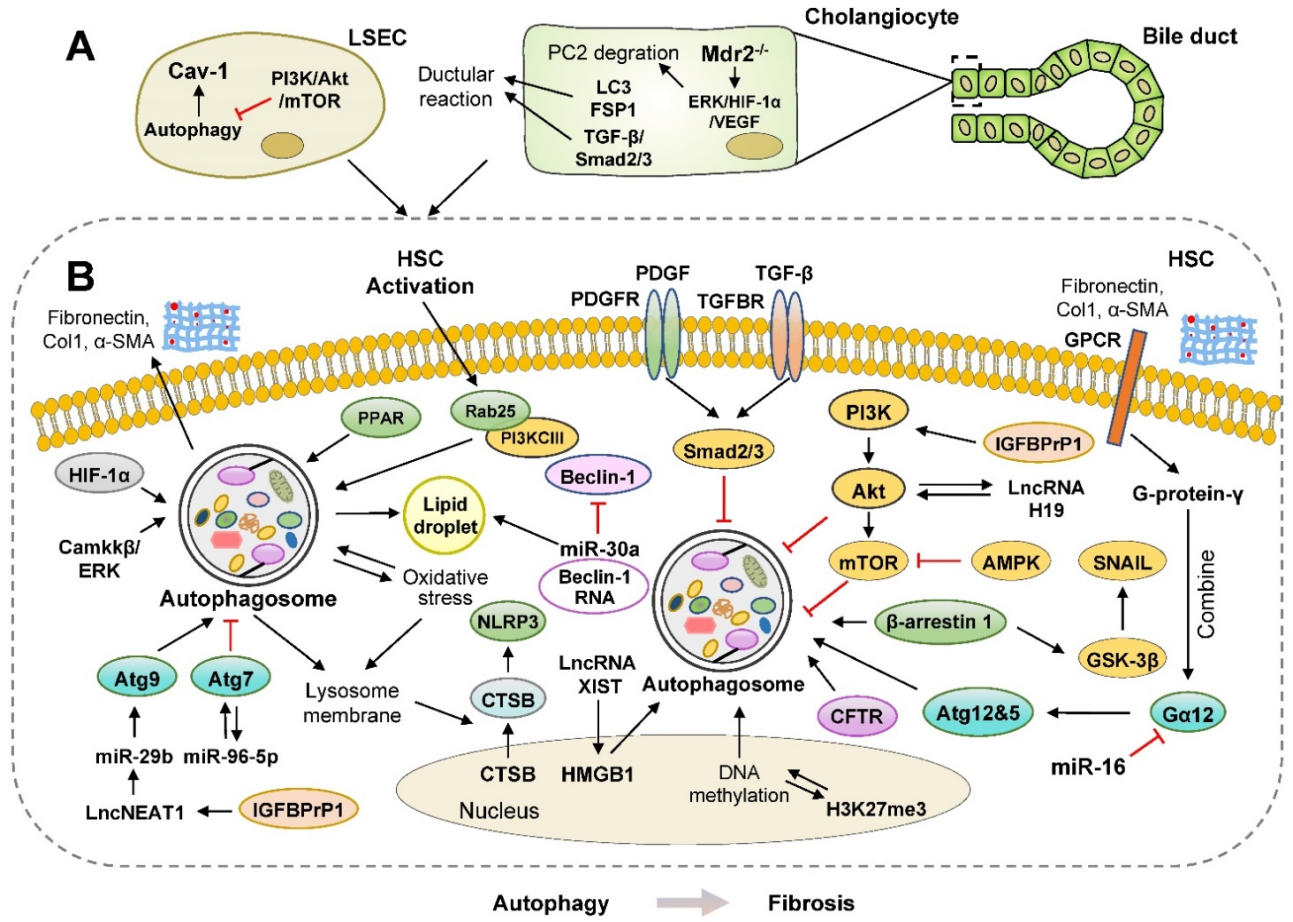
** Pro-fibrosis roles of autophagy in the liver.** Multiple studies have shown a positive correlation between activated autophagy and liver fibrosis. (A) Ductular reaction is usually associated with hepatic fibrogenesis. During ductular reaction, increased LC3-II is mainly located in cholangiocytes isolated from human and mouse fibrotic livers, which requires the activation of TGF-β/Smad2/3 signaling in an autophagy-dependent manner. Activated ERK/HIF-1α/VEGF pathways promote proteasomal PC2 degradation; however, blockade of autophagosome formation prevents PC2 degradation and ductular reaction. (B) The activation of autophagy promotes liver fibrosis by accelerating the digestion of LDs and upregulating the levels of several ncRNAs, including miR-96-5p, miR-29b, lncRNA XIST, lncRNA NEAT1 and lncRNA H19. During oxidative stress induced by ethanol or chemicals, autophagy is also rapidly activated, resulting in the elevated formation of NLRP3 inflammasomes and subsequent HSC activation. Furthermore, panel B represents how autophagy-related signaling pathways, including PI3K/Akt/mTOR, β-arrestin 1/GSK-3β/SNAIL, PDGF/TGF-β/Smads, promote liver fibrosis. AMPK: AMP-activated protein kinase; Akt: protein kinase B; LD: lipid droplet; mTOR: mammalian target of rapamycin; GPCR: G protein-coupled receptor; PDGFR: platelet-derived growth factor receptor; PPAR: peroxisome proliferator-activated receptor; TGFBR: transforming growth factor beta receptor.

**Figure 3:**
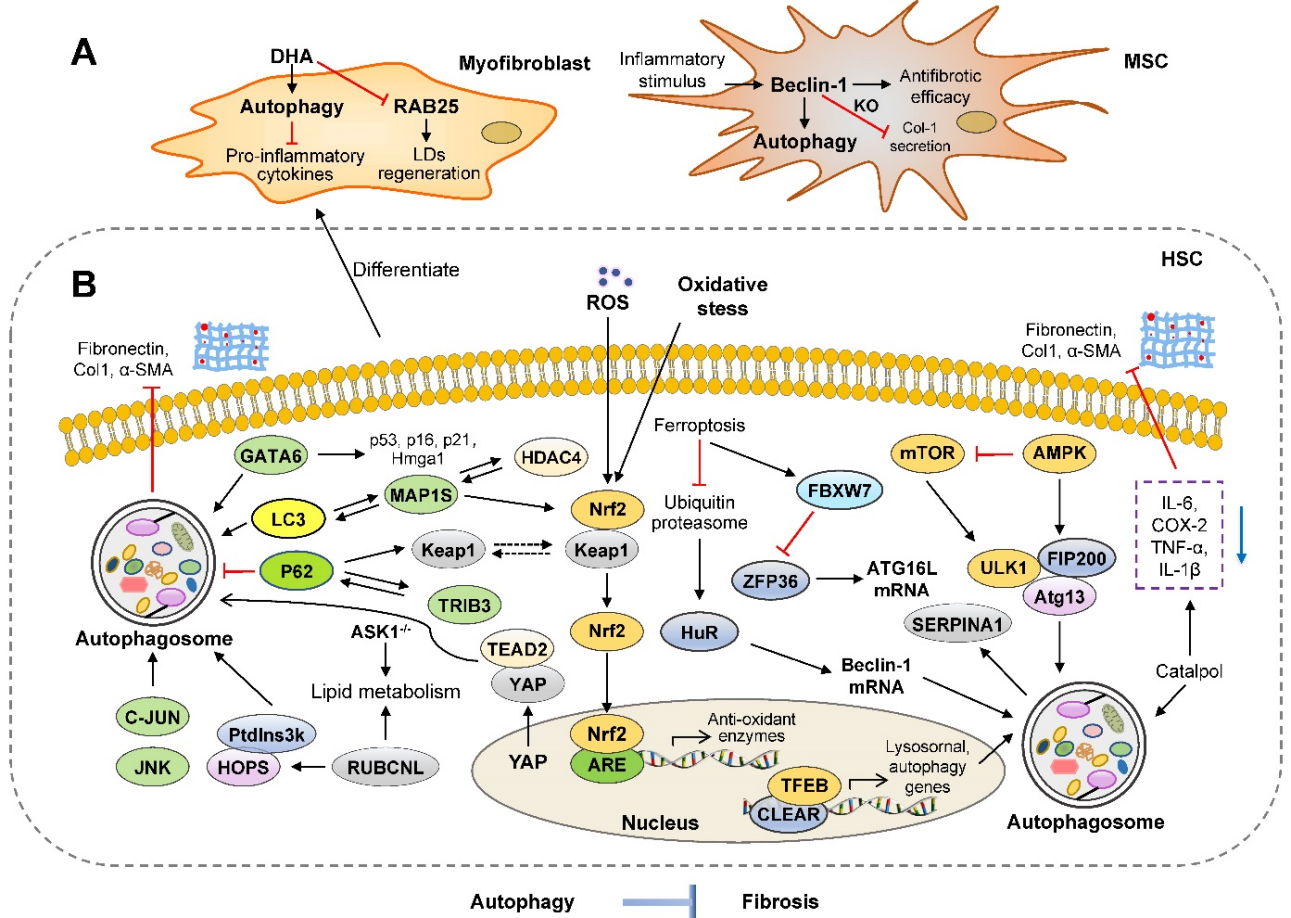
** Anti-fibrosis roles of autophagy in the liver.** Several studies suggest that the activation of autophagy may protect against liver fibrotic diseases through different mechanisms. (A) In myofibroblasts, the activation of autophagy inhibits liver fibrosis by promoting LD regeneration and downregulating pro-inflammatory cytokines. The knockdown of Beclin-1 suppresses autophagy and decreases collagen 1 secretion in mesenchymal stem cells (MSCs). (B) The upregulation of YAP, GATA6, MAP1S and complex of Ptdlns3k and HOPS activate autophagy and inhibit HSC activation. Furthermore, panel B represents how autophagy-related signaling pathways, including mTOR/ULK1, Nrf2/Keap1, JNK/c-JUN, protect against liver fibrosis. ARE: AU-rich element; JNK: c-Jun NH2-terminal kinases; HOPS: homotypic fusion and protein sorting; TFEB: transcription factor EB; HuR: human antigen R; RUBCNL: rubicon like autophagy enhancer; SERPINA1: serpin peptidase inhibitor, clade A 1; YAP: yes-associated protein.

It has been globally accepted that liver fibrosis is a common process of chronic or iterative liver diseases, such as hepatitis, cirrhosis, and other hepatic dysfunctions. Different types of hepatic cells, especially hepatic stellate cells (HSCs), are involved in the process of liver fibrosis 9, 10. Triggered by persistent liver parenchymal injury and the recruitment of immune cells, HSCs transform into active myofibroblasts and express α-SMA, vimentin and desmin. Myofibroblasts continuously produce ECM and collagen type I (Col-1) and secrete pro-fibrogenic cytokines, including transforming growth factor beta (TGF-β) 1 and tumor necrosis factor-alpha (TNF-α), into the hepatic microenvironment 11. Emerging evidence suggests that complicated interactions occur between autophagy and liver fibrosis, which has become a major focus of research** (Figure 2 and 3)**.

### Pro-fibrosis roles of autophagy in the liver

Multiple studies have shown a positive correlation between activated autophagy and liver fibrosis** (Figure 2)**. Cassidy *et al.* developed a mouse model incorporating a doxycycline (dox)-inducible shRNA system targeting Atg5 (Atg5i mice) and observed that the depletion of Atg5 induced by a dox-infused diet resulted in an abnormally large liver but showed no signs of fibrosis. Of note, hepatic fibrosis was observed in livers of Atg5i mice after autophagy restoration by changing the dox-infused diet to a standard diet for 6 weeks 12.

Recently, activated HSCs were observed to exhibit the features of autophagy in carbon tetrachloride (CCL_4_)- and thioacetamide (TAA)-induced liver fibrosis mouse model 13. In an alcoholic liver fibrosis mouse model, ethanol accelerated autophagy flux as indicated by downregulated p62 expression and upregulated LC3-II/LC3-I, which promoted oxidative stress and HSC activation 14. During oxidative stress induced by NaAsO_2_, autophagy was also rapidly activated and promoted the release of cytoplasmic CTSB from the nucleus into the cytoplasm following disruption of the lysosomal membrane. CTSB further mediated the formation of the NOD-like receptors containing pyrin domain 3 (NLRP3) inflammasome and activated rat primary HSCs and HSC-T6 cell lines 15. Recently, both hypoxia and lipopolysaccharide (LPS) induced autophagy in a hypoxia-inducible factor 1α (HIF-1α) dependent manner in LX-2 cells (human HSC lines), as indicated by the upregulation of the autophagy markers Atg3, p62 and LC3 16. Significant autophagy was also observed in a mouse model with combined diabetes and liver fibrosis. The researchers further suggested that acid-sensing ion channel 1α (ASIC1α) was crucial for the activation of autophagy in this model by regulating the Camkkβ/ERK pathway, and both pharmacological inhibition and genetic deletion of ASIC1α not only attenuated liver fibrosis in mice but also abrogated platelet-derived growth factor-β (PDGF-B)-induced HSC activation and proliferation 17. A previous study further demonstrated that in the hyperhomocysteinemia mouse model and HL-7702 cells (human normal hepatic cells), homocysteine inhibited cystic fibrosis transmembrane conductance regulator (CFTR) expression, activated autophagy and induced liver injury *via* interaction between histone H3 lysine 27 trimethylation (H3K27me3) and DNA methylation. The overexpression of CFTR inhibited the expression of Beclin-1, LC3-II/I and Atg12 and the formation of autophagosomes 18. Overall, by employing autophagy inhibitors, such as 3-Methyladenine (3-MA) and LY294002, or siRNAs targeting autophagic genes, the results from the above experimental models have verified that malfunction of autophagy obstructs the progression of liver fibrosis. Further investigations on how activated autophagy aggravates liver fibrosis by affecting multifarious signaling pathways are still required.

#### mTOR-mediated inhibition of autophagy

Mammalian target of rapamycin (mTOR) has been well established as a negative regulator of autophagy. AMP-activated protein kinase (AMPK) is an endogenous sensor of the AMP/ATP ratio and functionally maintains energy homeostasis. In general, AMPK activation triggers the inhibition of mTOR and thereby facilitates autophagy activation. However, it is noteworthy that 5-aminoimidazole-4-carboxamide-1-4-ribofuranoside (AICAR), a pharmacological activator of AMPK, inhibited autophagy flux and restricted the fibrotic potential by activating the protein kinase B (Akt) pathway in TGF-β-induced LX-2 cells 19. After TGF-β treatment, P62 aggradation was reduced, while autophagosomes, autolysosomes and autophagy flux were increased. Ursodeoxycholic acid (UDCA) inhibited autophagy flux and suppressed collagen aggradation in TGF-β-induced LX-2 cells and CCL_4_-induced mouse model 20. These UDCA-induced effects were completely abolished by an autophagy inducer, rapamycin, suggesting a crucial role of Akt/mTOR-mediated autophagy inhibition in the progression of liver fibrosis.

In addition, insulin-like growth factor binding protein-related protein 1 (IGFBPrP1), belongs to IGFBP family, is a novel TGF-β1-interacting profibrotic cytokine involved in the upregulation of TGF-β1 expression and ECM 21. Activated IGFBPrP1 promoted autophagy and activation of primary HSCs and HSC cell lines, as demonstrated by increasing long non-coding RNA (lncRNA) NEAT1/ microRNA-29b (miR-29b)/Atg9a axis and downregulating phosphatidylinositide3-kinase (PI3K)/Akt/mTOR signaling, which was verified by IGFBPrP1 overexpression or knockdown 22, 23. As expected, IGFBPrP1 expression and autophagy levels were observed to be concomitantly increased in bile duct ligation (BDL) mouse livers. This observation was further confirmed by Huang and colleagues that activated IGFBPrP1 stimulated autophagy and HSC activation in both IGFBPrP1 adenovirus-induced fibrotic mouse livers and JS-1 cells. Furthermore, the activation of lncRNA H19 promoted autophagy and HSC activation by interacting with the PI3K/Akt/mTOR pathway during IGFBPrP1-induced HSC activation 24. Caveolin-1 (Cav-1) is known as a structural protein that is associated with autophagy in liver sinusoidal endothelial cells (LSECs). Autophagic degradation of Cav-1 and F-actin remodeling were increased in CCL_4_-induced LSEC defenestration and liver fibrosis by inhibiting the NO-dependent PI3K-Akt-mTOR pathway. Additionally, inhibition of autophagy, including 3-MA, bafilomycin and Atg5-siRNA treatment, alleviated Cav-1 depletion and maintained LSEC fenestrae 25.

#### Autophagy and digestion of lipid droplets

Autophagy regulates lipid accumulation by maintaining the balance between LD synthesis and degradation. Physiologically, quiescent HSCs are fat-storing cells (FSCs) and vitamin A-storing cells, whereas under pathological conditions, activated HSCs lose lipid droplets and vitamin A. The activation of autophagy accelerates the catabolic process of LDs in transforming HSCs, leading to the release of free fatty acids (FFAs), adenosine triphosphate (ATP) and triglycerides (TG), which provide extra energy for HSC activation. Both 3-MA, a chemical inhibitor of autophagy, and transfection with siRNAs targeting Atg3 and Atg7 inhibited autophagy in HSCs and in the livers of CCL_4_- or TAA-treated mice and reduced lipid metabolism and energy production, which then inhibited ECM production and liver fibrosis 13. Another study further suggested that LD disappearance during HSC activation is associated with increased autophagy. HSC activation was shown to promote the autophagic recognization, wrapping and elimination of LDs by triggering the overexpression of Rab25 and accelerating the binding of Rab25 to PI3KCIII. Antioxidants, such as glutathione and N-acetyl cysteine, inhibited Rab25-induced autophagy and increased intracellular LDs, which subsequently arrested HSC activation 26. Atg2A was recently found to be localized on LDs, and its release was required for autophagosome formation and HSC activation. Compared to the changes in LDs in activated HSCs, Atg2A deficiency resulted in enlarged LDs, suppressed autophagy flux and decreased HSC activation in LX-2 cells 27. Additionally, Beclin-1 downregulation, mediated by the binding of miR-30a to its 3'-UTR regions, inhibited HSC autophagy, increased lipid accumulation and decreased the expression of α-SMA and Col-1 in the livers of BDL-induced fibrotic mice. Consistently, knockdown of Beclin-1 using siRNA simultaneously prevented HSC activation and autophagy in LX-2 cells 28.

LPS, another stimulator for HSC activation, obviously increased autophagy flux, reduced the number of LDs and lipid staining areas in HSCs and CCL_4_-induced mice, which was attenuated by autophagy inhibitor chloroquine (CQ) or silencing Atg5 29. Peroxisome proliferator-activated receptor α (PPARα) plays a crucial role in regulating fatty acid transport and β-oxidation, lipid metabolism and inflammation in livers. After arsenic trioxide (As_2_O_3_) treatment, HSCs were activated by reducing LD levels and activating the PPARα and autophagy pathways 30.

#### Interaction between autophagy and the TGF-β pathway

After TGF-β stimulation, phosphorylated Smad2/3 bind to Smad4 to form a Smad2/3/4 complex and then translocate into the nucleus, which regulates gene expression and induces HSC activation. PDGF-B is documented as an essential contributor to the phosphorylation of Smad2/3 by binding to its receptor PDGFR and synergizing with TGF-β-mediated profibrotic signaling. Recently, it has been reported that quercetin and salidroside reduced autophagy and suppressed liver fibrosis by increasing PI3K/Akt signaling pathway and alleviating TGF-β/Smads signaling pathways in a BDL-induced mouse model, respectively 31, 32. Consistently, isorhamnetin blocked autophagy and inhibited HSC activation in different fibrotic mouse models by downregulating the p38 mitogen-activated protein kinase (MAPK) and TGF-β1-mediated Smad3 signaling pathways 33. Furthermore, *B. abortus* infection induced HSC activation with a fibrogenic phenotype and promoted the activation of autophagic pathways in HSCs. On the other hand, activated autophagy by rapamycin also increased the *B. abortus*-mediated the secretion of TGF-β1 and collagen deposition in HSCs, which was reversed by a lysosomal protease inhibitor 34.

Recently, it has been reported that β-arrestin 1 was increased in human and mouse fibrotic livers and promoted autophagy by recruiting Beclin-1 in the presence of TGF-β signaling. β-arrestin 1 knockout (KO)- or 3-MA treatment-mediated autophagy inhibition led to an accumulation of glycogen synthase kinase-3β (GSK-3β), which phosphorylated Snail and decreased its nuclear translocation. On the other hand, by facilitating TGF-βRII, β-arrestin 1 promoted autophagy, rescued Snail by inhibiting GSK-3β and thus promoted Snail-dependent HSC growth and activation 35.

#### Non-coding RNAs and autophagy

The regulatory role of other newly discovered non-coding RNAs involved in autophagy has gradually attracted a great deal of attention. High-mobility group box-1 (HMGB1) is a nuclear DNA-binding protein and DAMP that is implicated in innate immunity. Recently, researchers found that the overexpression of HMGB1 increased the number of autophagic vacuoles, upregulated levels of autophagy marker LC3-II and induced liver fibrosis, which was alleviated in LX-2 cells transfected by LC3-siRNA 36. As with other lncRNAs, lncRNA XIST functioned as a competitive endogenous RNA (ceRNA) and harbored putative binding sites of miR-29b, which directly targeted and inhibited the expression of HMGB1. Hence, lncRNA XIST upregulated HMGB1 expression, promoted ethanol-induced autophagy and eventually activated HSCs 37. G protein-coupled receptors (GPCRs) play complex roles in cellular signal transduction in HSC activation. Both G protein-γ, ligands of GPCRs coupling with Gα12, and Gα12 were overexpressed in HSCs and fibrotic livers and facilitated autophagy by promoting Atg12-5 formation. Considering as a direct inhibitor of Gα12, miR-16 was abundant in quiescent HSCs, while disordered miR-16 led to HSC activation by triggering Gα12 accumulation-related autophagy 38. Furthermore, Yu *et al.* first identified that miR-96-5p was an abnormally upregulated miRNA in fibrotic tissues. By using computational predictions and luciferase assays, they suggested that miR-96-5p directly interacted with Atg7, which then blocked autophagy and inhibited HSC activation. After reintroducing Atg7, miR-96-5p mimics-mediated suppression of α-SMA, Col-1 and autophagy activity were completely reversed in LX-2 cells 39.

#### Autophagy in ductular reaction

Ductular reaction (DR), characterized by intensive bile duct proliferation, is usually associated with persistent inflammation and fibrosis. Recently, accumulating evidence has suggested that autophagy plays a critical role in DR and the fibrogenesis in cholangiopathies. A previous study demonstrated that compared with non-cirrhotic livers, co-localization of LC3-II and lysosome-associated membrane protein-1 (LAMP-1), increased levels of LAMP-2 and enhanced maturation of lysosomal cathepsin D were detectable in cirrhotic livers. Besides, by using immunofluorescence staining of cytokeratin 19 (CK-19), Hung *et al.* illustrated that in human cirrhotic livers and AAF- and CCL_4_-induced mouse livers, increased LC3-II was primarily located in cholangiocytes during DR, which was markedly attenuated by CQ inhibitor, suggesting that autophagy was positively correlated with DR in cirrhosis 40. They further demonstrated that ductular cells from fibrotic livers exhibited increased autophagy and underwent a mesenchymal transition, which required the activation of TGF-β/Smad2/3 signaling in an autophagy-dependent manner. Importantly, ductular cells were not only positive for LC3-II but also showed increased expression of TGF-β and fibroblast-specific protein-1 (FSP1) in cirrhotic human livers 41. As a nonselective calcium channel, the downregulation and loss of function of polycystin-2 (PC2) is associated with polycystic liver diseases, characterized by the formation of multiple cysts and fibrotic scars. In multidrug resistance gene 2 (Mdr2) KO, BDL- and 3,5-diethoxycarbonyl-1,4-dihydrocollidine-induced mouse model, activated extracellular signal-regulated kinase 1/2 (ERK1/2)/HIF-1α/vascular endothelial growth factor (VEGF) pathways promoted proteasomal PC2 degradation and downregulated PC2 protein expression. Researchers further confirmed these *in vivo* observations in proinflammatory cytokine-, nitric oxide donor- and endoplasmic reticulum (ER) stressor-induced primary mouse cholangiocytes. Consistently, blockade of autophagosome formation by PI3K inhibitors and CQ or inhibition of proteasomes with bortezomib prevented PC2 degradation, DR and liver fibrosis 42. These studies suggest the potential involvement of autophagy in DR and liver injury, but further investigation is urgently required to assess this possibility.

### Anti-fibrosis roles of autophagy in the liver

Several studies suggest that activation of autophagy may protect against liver fibrotic diseases through different mechanisms (**Figure 3**). In contrast with Atg5 knockdown results from Cassidy 12, Ni *et al.* generated liver-specific Atg5 KO (L-Atg5 KO) mice, L-Atg5/mTOR, and L-Atg5/Raptor double knockout (DKO) mice and further found that L-Atg5/mTOR DKO and L-Atg5/Raptor DKO mice had increased hepatic inflammation and fibrosis at 6 months of age and even developed liver tumors at 9 months of age, suggesting the importance of autophagy in the progression of liver fibrosis 43. Furthermore, in Atg5 liver-specific KO mice, profibrotic genes, including Col-1, α-SMA, connective tissue growth factor (CTGF), and TGF-β were increased in hepatocytes 44. In the wild type (WT) mice, Src inhibitors (saracatinib and PP2) markedly suppressed the expression of α-SMA in HSCs and decreased the TGF-β-induced CTGF expression by increasing autophagy flux in hepatocytes. However, in primary hepatocytes of hepatocyte-specific Atg7 KO mice (Atg7^f/f^ Alb-Cre^+^), Src inhibitors-induced autophagy and increased CTGF levels did not occur 45. Thus, there is still a substantial gap between the current understanding of the potential roles of autophagy deficiency in the progression of liver fibrosis and whether Atg5 and Atg7 can be used as effective targets for the treatment of liver diseases.

#### Autophagy and liver fibrosis mediated by multiple pathways

The crosstalk between autophagy and HSC activation determines the fibrotic process and is governed by multiple signaling pathways. Yes-associated protein (YAP) is responsible for cell proliferation and angiogenesis through regulation of TEAD family transcription factors. In the BDL-induced mouse model and rat primary HSCs, dihydrotanshinone increased autophagy flux and suppressed ECM accumulation by inhibiting YAP nuclear translocation, preventing the formation of YAP and TEAD2 complex and downregulating fibrogenic genes, including survivin and CTGF 46. Among Rab GTPase family proteins, Rab18 is known as a mediator of stellate cell activation. A previous study demonstrated that Rab18 downregulated autophagy flux and inhibited HSC activation in LX-2 cells, which was further illustrated by Rab18 knockdown, overexpression and pharmacologic inhibition analyses 47. In addition to cell proliferation and activation, other signaling pathways have been shown to play crucial roles in regulating autophagy-associated HSC senescence and fibrosis. It has been reported that docosahexaenoic acid (DHA) treatment promoted autophagy flux and senescence of activated HSCs in rat fibrotic livers and increased the expression of senescence markers including p53, p16, p21 and HMGa1 by promoting GATA binding protein 6 (GATA6) accumulation in LX-2 cells. Furthermore, treatment with various autophagy inhibitors, such as 3-MA and CQ, prevented DHA-induced p62 degradation, which in turn inhibited GATA6 accumulation and HSC senescence 48.

Several studies reported that microtubule-associated protein 1S (MAP1S) interacted with LC-3 and positively promoted autophagy flux. Driven by binding with LC3, fibronectin was translated and the resulting protein was exported to the cell surface for its assembly. Subsequently, fibronectin was degraded depending on autophagy and lysosomal activities. Notably, in LC3-overexpressing MAP1S KO mice, overexpression of LC3 enhanced the synthesis of fibronectin and MAP1S depletion caused the impairment of autophagy flux, synergistically resulting in fibronectin accumulation and aggravated liver fibrosis 49. Upon exposure to oxidative stress, Nrf2 forms a complex with Keap 1 and regulates the transcription of cytoprotective enzymes by binding to antioxidant response element (ARE). A previous study further suggested that MAP1S stability was also regulated by its interaction with histone deacetylase (HDAC4). Spermidine, a non-canonical Nrf2 inducer, increased MAP1S stability and promoted autophagy flux through depletion of cytosolic HDAC4, which in turn reduced liver fibrosis and extended lifespan in MAP1S-deficient mice 50. In addition to the selective degradation of ubiquitinated cargo, p62 is involved in the degradation of Keap1 51. MAP1S was also reported to enhance Nrf2 stabilization, compete with Keap1 for combining to Nrf2 and eventually promote the degradation of Keap1 by inducing autophagy. Consistently, spermidine suppressed liver fibrosis by activating Nrf2 and increasing MAP1S-mediated autophagy, which was partly reversed in Nrf2 or p62 single KO mice and completely inhibited in Nrf2 and p62 DKO mice 52. These observed anti-fibrotic impacts of MAP1S rely upon the promotion of autophagy flux, once again, showing the importance of increased autophagy flux in the development of liver fibrosis.

Recently, it has been reported that high levels of tribbles pseudokinase 3 (TRIB3) and p62 were positively correlated in livers of patients with cirrhosis. Liver-specific knockdown of TRIB3 restored autophagy activity in livers and protected the liver against TAA- and BDL-induced liver fibrosis. These findings further demonstrated that TRIB3 interacted with p62, hindered its binding to LC3 complexes and further resulted in p62 accumulation and impaired autophagy flux, eventually leading to decreased late endosome degradation and accelerated INHBA/Activin A-enriched exosomes-caused HSC activation and proliferation 3. A recent study demonstrated that in liver fibrosis, fibrogenic extracellular vesicles (EVs), including exosomes, induced HSC migration in an autocrine manner. Unlike TGF-β stimulation, PDGF and its downstream molecule Src homology 2-containing protein tyrosine phosphatase 2 (SHP2) exerted a pro-fibrotic effect in HSCs by enhancing the release of fibrogenic EVs *via* autophagic inhibition and activation of the mTOR pathway 53.

Farnesoid X receptor (FXR), known as a bile acid nuclear receptor, has been reported to be involved in HSC activation and autophagy regulation. In addition, probucol inhibited LPS- and CCL_4_-induced activation of HSCs both *in vitro* and* in vivo* by increasing the expression of FXR and accelerating autophagy, which was verified using the FXR antagonist Z-guggulsterone, and the FXR agonist GW4064 54. It has been reported that iron overload accelerated liver damage by reducing the expression of LC3-II, LC3-I and Beclin-1 and inhibiting the activation of Atg3, 5, 7 and 12 in ethanol- and high-fat diet (HFD)-induced mouse model 55. Unc-51-like kinase 1 (ULK1) forms a protein complex with FIP200 and the autophagy protein Atg13 to mediate the initiation of autophagy and is regulated by the mTOR and AMPK pathways 56. Recent studies reported that norUDCA improved alpha-1-antitrypsin (α1AT) deficiency-induced accumulation of a1AT mutant Z and liver fibrosis in mice by promoting autophagy-mediated degradation of a1ATZ protein and activating the AMPK/ULK1 signaling pathway, which could be abolished by CQ and the ULK1 inhibitor SBI-0206965 57, 58. Transcription factor EB (TFEB) is a key transcriptional factor driving lysosomal gene expression and is the primary player involved in coordinating the formation of autophagosomes and autophagosome-lysosome fusion 59. Serpin peptidase inhibitor, clade A (SERPINA1) deficiency is a common genetic cause of chronic liver disease. Liver-directed gene transfer of TFEB attenuated hepatic fibrosis by accelerating the autophagy-dependent degradation of SERPINA1 polymer in autolysosomes and decreasing SERPINA1 monomer expression in SERPINA1 deficient mice 60. Furthermore, Liu *et al.* reported that platycodin D mediated the activation of autophagy and suppressed activation of HSCs by promoting phosphorylation of C-Jun NH2-terminal kinases (JNK) and C-Jun, which was reversed by JNK inhibitor P600125 61.

#### Autophagy and lipid accumulation in hepatic cells

Although a previous study reported that autophagy contributed to the recognization, wrapping and elimination of LDs by upregulating Rab25 expression in HSCs 26, a more recent study identified that the exact role autophagy played in LDs was dependent on specific cell types and different stimuli. Unlike quiescent HSCs, DHA increased autophagosome generation and autophagy flux, however, it paradoxically induced LD regeneration in activated HSCs and improved liver fibrosis by inhibiting Rab25 expression 62.

Interestingly, an increasing number of studies have pointed out that the induction of autophagy may offer benefits to fibrotic livers, probably because it alleviates steatosis without directly targeting the process of liver fibrosis itself. Autophagy dysfunction-associated p62 accumulation is also regarded as one of the histological features of non-alcoholic fatty liver disease (NAFLD) and non-alcoholic steatohepatitis (NASH). The number of hepatocytes with p62 aggregation was positively correlated with an increased number of autophagosomes, the M1-polarization of macrophages and liver fibrosis in livers from NAFLD patients 63. Silent information regulator 1 (SIRT1), an NAD^+^ dependent histone deacetylase, regulated energy and lipid homeostasis in the liver, and miR-34a-5p was the direct inhibitor of SIRT1. Melatonin reversed HFD-caused liver fibrosis and NAFLD by increasing the ratio of LC3-II/I, decreasing cytoplasmic p62 levels and reducing the expression of miR-34a-5p in WT, but not SIRT1 heterozygous (HET) mice 64. Similar to previous results, the activation of autophagy by rapamycin alleviated methionine choline-deficient (MCD) diet-caused liver fibrosis in a NASH mouse model 65. Interestingly, ASMase KO mice were resistant to HFD-induced hepatic ER stress, fibrosis and NASH but sensitive to that induced by tunicamycin. Impaired autophagy, lower autophagy flux and increased ER stress were observed in primary mouse hepatocytes from ASMase KO mice 66. Rubicon like autophagy enhancer (RUBCNL/Pacer) is a vertebrate-specific autophagy regulator that contributes to the formation of autophagosomes. Previous studies demonstrated that homotypic fusion and protein sorting (HOPS) complexes had an initial recognition and fusion capabilities in the process of membrane fusion. Activated RUBCNL promoted autolysosome formation by engaging the class III phosphatidylinositol 3-kinase (PtdIns3K) and HOPS complexes in the cytoplasm 67. Cheng *et al.* further demonstrated that RUBCNL acetylation markedly promoted HOPS complex recruitment, caused autophagosome maturation and lipid metabolism. Additionally, hepatocyte-specific RUBCNL KO mice exhibited impaired autophagy flux, increased LD accumulation and severe liver fibrosis 68, 69. It has been identified that apoptosis signal-regulating kinase 1 (ASK1) was a critical suppressor of NASH and liver fibrosis. HFD- and chow-fed liver-specific ASK1-KO mice developed more severe hepatic steatosis and fibrosis due to blunted autophagy. Consistently, in hepatocytes, depletion of ASK1 impaired autophagy and elevated lipid accumulation. On the other hand, conditional liver-specific ASK1 overexpression protected mice against HFD- and CCL_4_-induced liver fibrosis. Remarkably, in lean and obese human livers, the expression of ASK1 was positively correlated with the autophagy marker LC3-II and negatively associated with liver fat content and NASH scores 70.

#### Autophagy and RNA-binding proteins

Emerging studies demonstrated that human antigen R (HuR), an RNA-binding protein, plays a critical role in ferroptosis and liver fibrosis 71. After exposing HSCs to ferroptosis stimulators, HuR was significantly increased by inhibiting the ubiquitin-proteasome pathway and enhanced classical ferroptosis through increased autophagosome generation and autophagy flux in HSCs. This treatment also improved BECN1/Beclin-1 mRNA stability and accelerated the activation of autophagy by binding the AU-rich elements (AREs) within the F3 of the 3'-untranslated region of Beclin-1 mRNA, which was prevented by internal deletion of the F3 region 72. Additionally, HSC-specific knockdown of HuR impaired sorafenib-promoted HSC ferroptosis, and finally, aggravated liver fibrosis in mice. More recently, another RNA-binding protein, ZFP36 ring finger protein (ZFP36) was reported to play a crucial role in regulating ferroptosis in HSCs. After treatment with ferroptosis-inducing compounds, the ubiquitin ligase F-box and WD repeat domain containing 7(FBXW7)/CDC4 reduced the expression of ZEP36 by recognizing the SFSGLPS motif in HSCs. Interestingly, the overexpression of ZEP36 not only inhibited autophagy activation by destabilizing ATG16L1 mRNA but also promoted ATG16L1 mRNA decay through binding to AREs within its 3'-untranslated region. Consistently, the overexpression of ZEP36 prevented erastin- and sorafenib-induced HSC ferroptosis and aggravated liver fibrosis. Clinically, fibrotic patients with HCC receiving sorafenib monotherapy showed lower expression of ZEP36 and ferroptosis activation 73. In accordance with the above findings, it has been revealed that autophagy-dependent ferroptosis in HSCs functions as a potential target for the treatment of liver fibrosis.

#### Autophagy and anti-inflammatory effects

Correlative evidence has provided insights into the anti-fibrotic role of autophagy under inflammatory conditions. In response to inflammatory stimuli including cytokines TNF-α, interferon gamma (IFN-γ) and TGF-β1, Beclin-1 was increased and then triggered autophagy in mesenchymal stem cells (MSCs). Knockdown of Beclin-1 not only suppressed autophagy but also promoted the antifibrotic efficacy of MSCs and decreased collagen 1 secretion 74. Although autophagy in macrophages promoted the production of inflammatory cytokines, especially interleukin 1α (IL-1α) and IL-1β during chronic liver injury, Atg5 deficiency aggravated CCl_4_ administration-triggered excessive release of cytokines associated with increased ECM accumulation and liver fibrosis. Conditional cultured medium of macrophage isolated from Atg5 KO mice contained more ROS-induced IL-1α and IL-1β and upregulated profibrogenic gene expression in hepatic myofibroblasts 75. As expected, DHA increased autophagy flux and autophagosome generation in activated HSCs and thus suppressed the expression of pro-inflammatory cytokines, including IL-1β, IL-6 and TNF-α, in the activated HSCs and inhibited inflammation in CCL_4_-induced rat model 76. It has been indicated that catalpol upregulated the expression of autophagy markers, such as LC3-II and Beclin-1, and reduced the expression of α-SMA and Col-1 and decreased ECM accumulation by downregulating the levels of the inflammatory cytokines, IL-1β, TNF-α, IL-18, IL-6 and cyclooxygenase-2 (COX-2) in CCL_4_-induced rat models. Furthermore, the anti-inflammatory and anti-fibrotic effects of catalpol toward HSCs were abolished by autophagy inhibitor LY294002 or Atg5-siRNA 77.

## Lung fibrosis and autophagy

**Figure 4:**
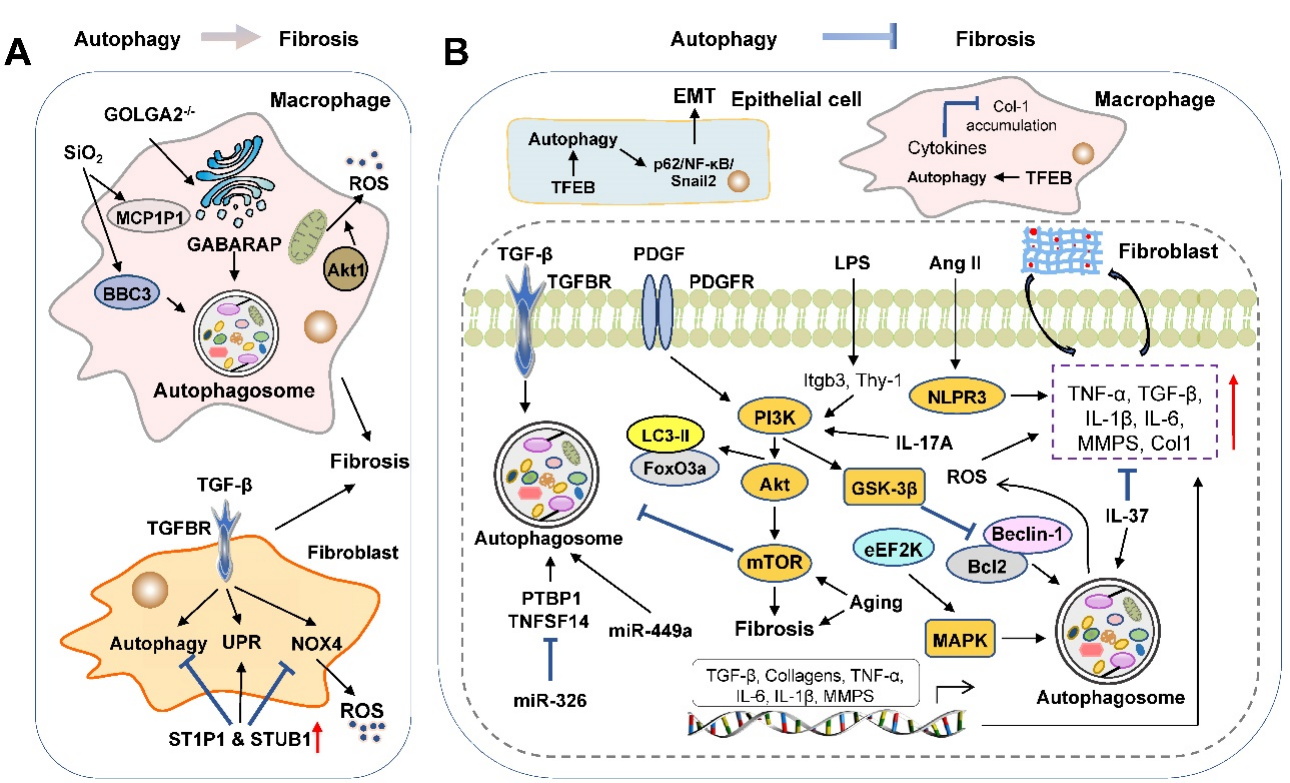
** Molecular targets of autophagy in regulating lung fibrosis.** Advances have been achieved in our understanding of dual roles of autophagy in pulmonary fibrosis. (A) In macrophages, the upregulation of BBC3 and MCP1P1 and GABARAP release promote the activation of autophagy and fibrosis. In addition, UPR and the release of ROS induced by TGF-β contribute to the activation of lung fibroblasts. (B) Accumulating evidence suggests that the activation of autophagy improves lung fibrosis through several signaling pathways, including NLPR3, GSK-3β, PI3K/Akt/mTOR and MAPK. Furthermore, several microRNAs have also been demonstrated to perform essential functions in regulating lung fibrosis. Increased expressions of miR-326 and miR-449α promote autophagy activity of fibroblasts and alleviate lung fibrosis. eEF2K: eukaryotic elongation factor-2 kinase; PTBP1: polypyrimidine tract-binding protein; Sio2: silicon dioxide; TNFSF14: tumor necrosis factor superfamily-14; UPR: unfolded protein response.

Idiopathic pulmonary fibrosis (IPF), known as a chronic and progressive fibro-proliferative lung disease, leads to severe breathlessness, respiratory failure and even death. Alveolar epithelium injury, differentiation of resident fibroblast-to-myofibroblast and accumulation of myofibroblast, which further results in ECM deposition and lung fibrosis, are involved in the process of IPF 78. Interestingly, advances have been achieved in our understanding of dual roles of autophagy played in pulmonary fibrosis **(Figure 4)**.

### Pro-fibrosis roles of autophagy in the lung

It is well established that autophagy flux is involved in the progression and severity of lung fibrosis. Fibroblast activation during lung fibrosis is driven not only by the release of cell contents and ROS from epithelial cells but also by pro-inflammatory mediators secreted by macrophages. Current studies demonstrated that activated macrophages might contribute to lung fibrosis by activating autophagy. After treatment with silica, increased monocyte chemotactic protein-1-induced protein 1 (MCP1P1) facilitated macrophage apoptosis and promoted the proliferation and migration of fibroblasts by activating autophagy, which was reversed by 3-MA 78. Furthermore, silica increased the autophagy and cell apoptosis of alveolar macrophages by elevating the expression of BCL-2 binding component 3 (BBC3) in the mouse model, which was significantly enhanced by rapamycin and was mitigated by BBC3-siRNA and 3-MA 79.

Unfolded protein response (UPR) is responsible for restoring defective proteostasis and attenuating protein synthesis upon ER stress, which in turn promotes the capacity of ER in misfolded protein processing. There have been significant advances in understanding the role of dysregulated autophagy flux and UPR in various human fibrotic diseases during the last decade. TGF-β-induced autophagy flux, as illustrated by LC3B accumulation and p62 degradation, UPR and ECM accumulation were significantly suppressed by treating with bafilomycin A1, an autophagy flux inhibitor in primary lung fibroblasts 80. NADPH oxidase 4 (NOX4)-induced ROS production is involved in TGF-β-mediated cell differentiation and apoptosis. The previous study demonstrated that the treatment of azithromycin was reported to inhibit TGF-β-induced NOX4 and myofibroblast differentiation in the human lung fibroblasts. Furthermore, it inhibited autophagy flux and enhanced UPR by increasing the expression of STIP1 homology and U-box containing protein 1 (STUB1) and promoting NOX4 ubiquitination 81.

Furthermore, some recent researches applied KO animals to investigate the relationship between autophagy and pulmonary fibrosis. In addition, previous studies also demonstrated that Akt1 suppressed cell apoptosis, induced mitochondrial ROS and mitophagy in macrophages and promoted lung fibrosis. Deletion of macrophage Akt1 kinase was observed to induce impaired mitophagy and reduce TGF-β expression in the macrophages (Akt1^-/-^Lyz2-cre) and Parkin 2 (PARK2) KO mice 82. Phosphoglycerate mutase family member 5 (PGAM5), a mitochondrial protein, regulates mitochondrial homeostasis and participates in lung fibrosis. In PGAM5 KO lung fibrosis mouse model, pulmonary fibrosis and mitophagy were reduced compared to the PGAM5-proficient control 83. The formation of autophagosome is initiated from the assembly of phagophore and Golgi serves as a double-membraned source of the phagophore. It has been reported that the deletion of Golgin A2 (GOLGA2), encoding a cis-Golgi protein, induced the formation of the autophagosome *via* the releases of GABARAP from the Golgi 84. Recently, researchers have revealed that the deletion of GOLGA2 induced autophagy and lung fibrosis by disrupting the Golgi function, increasing alveolar macrophages and reducing subcellular lipid storage in GOLGA2 KO mice 85.

### Anti-fibrosis roles of autophagy in the lung

Emerging evidence supports that the dysfunction of autophagy promotes the progression of IPF. Kesireddy and colleagues generated LC3B KO mice and observed that LC3B KO mice displayed increased lung epithelial cell apoptosis, ER stress and susceptibility to bleomycin (BLM)-induced lung fibrosis 86. Furthermore, after BLM administration, Atg4b deficient mice displayed a higher inflammatory response and severe lung fibrosis, associated with the increased production of proinflammatory cytokines, neutrophilic infiltration, alveolar and bronchiolar epithelial cell apoptosis and collagen accumulation 87. Reduced autophagy flux, altered mitophagy and defected mitochondrial function were also observed in BLM- and TGF-β-induced IPF mouse models as well as TGF-β-mediated fibroblast differentiation *in vitro* model 88. Additionally, in a silica nanoparticle (SiNPs)-induced IPF model, after engulfed by alveolar epithelial cells, SiNPs inhibited autophagy flux, induced autophagosome accumulation and then promoted IPF by suppressing lysosomal degradation and promoting cyclic-3',5'-adenosine monophosphate (cAMP) in IPF mouse model. Enhanced autophagy flux by rapamycin improved lung fibrosis in these mice 89.

Plenty of studies have identified that the proliferation, migration, invasion and EMT of fibroblasts play a crucial role in the progression of IPF. Protein phosphatase 1 regulatory subunit 13B (PPP1R13B), a major member of the apoptosis-stimulating protein of p53 family, was upregulated and its upstream circRNA-012091 was downregulated in silica-treated fibroblasts. The overexpression of PPP1R13B promoted the proliferation and migration of lung fibroblasts by increasing ER stress and autophagy, which was reversed by 3-MA 90. Intemperate and pathogenic invasiveness of lung fibroblasts is a representation of IPF that has been increasingly recognized and studied over the last decades. Inhibition of vimentin intermediate filaments (VimIFs) was shown to suppress the invasiveness of fibroblasts and further protect mice from fibrosis. Specifically, with the treatment of a VimIF inhibitor, withaferin (WFA), autophagy was increased and invasiveness of fibroblasts was restricted in the BLM-induced mouse model and in the 3D lung organoids and pulmospheres derived from IPF patients 91. Most recently, it was revealed that the expressions of signal transducer and activator of transcription 3 (STAT3) and Janus kinase 2 (JAK2) were upregulated in the lung tissue of IPF patients. Dual inhibition of p-JAK2/p-STAT3 ameliorated BLM-induced lung fibrosis and collagen deposition by preventing fibroblast to myofibroblast transition and fibroblast migration and ameliorating impaired autophagy 92. Furthermore, it has been shown that Snail superfamily, including snail1, snail2 and snail3, promoted EMT process by repressing the levels of intracellular E-cadherin. Notably, inhibition of autophagy by HCQ accelerated EMT by increasing the p62-nuclear factor κB (NF-κB)-Snail2 pathway in alveolar epithelial cells 93.

Similar to findings in liver fibrosis, many studies have consistently indicated that TFEB activation and nuclear translocation were closely associated with autophagy and rescued fibrotic diseases. Annexin A2 (ANXA2) was observed to alleviate lung fibrosis caused by BLM by combining with BLM and accelerating TFEB-mediated autophagy flux *in vitro* and *in vivo*, which was abolished by CQ and bafilomycin A1 in the ANXA2 genetic depletion mice and CRISPR-Cas9-engineered ANXA2 mutation lung epithelial cells 94. More recently, it has been reported that the overexpression of TFEB or treatment with a TFEB activator, trehalose, suppressed the levels of inflammatory cytokine and prevented lung fibrosis by attenuating lysosomal dysfunction and augmenting autophagy flux in alveolar macrophages, which was abolished by CQ, bafilomycin A1 and TFEB knockdown 95. Collectively, these findings support that the impairment of autophagy in IPF progression is tightly regulated by the interplay between TFEB location and lysosomal dysfunction.

Synthesized by Sphks, sphingosine-1-phosphates (S1Ps) act on the S1PR receptor and S1P signaling plays a critical role in the progression of IPF. It has been demonstrated that S1P lyase (S1PL) was increased in the BLM-induced mouse model and patients with IPF. The overexpression of S1PL suppressed expression of α-SMA and fibronectin and S1P-induced fibroblast differentiation by upregulating the expression of LC3 and Beclin-1 and inhibiting Smad3 expression. Consistent with these results, the knockdown of S1PL in mice augmented BLM-induced pulmonary fibrosis 96. Eukaryotic elongation factor-2 kinase (eEF2K), a negative modulator of protein synthesis, regulates the process of autophagy under environmental stress. A recent study reported that eEF2K activated autophagy and ameliorated the activation of lung myofibroblasts *via* upregulating P38 MAPK signaling, which was abolished by a P38 MAPK inhibitor SB203580 and eEF2K-siRNA transfection 97.

Notably, several microRNAs have also been demonstrated to perform an essential function in regulating lung fibrosis. Previous studies have identified that miR-449α was significantly reduced in the silica-induced mouse model and TGF-β treated fibroblasts. The overexpression of miR-449α alleviated lung fibrosis through the upregulation of autophagy activity both *in vitro* and *in vivo* 98. Furthermore, the level of miR-326 was down-regulated in silica-induced pulmonary fibrosis mouse, silicon monoxide-stimulated lung epithelial cells, HBE and A549, and TGF-β-treated lung fibroblast cells, MRC-5 and NIH/3T3. Increased expression of miR-326 promoted autophagy activity of fibroblasts and alleviated silica-induced pulmonary fibrosis by downregulating the expressions of polypyrimidine tract-binding protein (PTBP1) and tumor necrosis factor superfamily-14 (TNFSF14) 99.

#### Autophagy and the PI3K-Akt-mTOR pathways

Previously, researchers have shown that activated M-CSF receptor (M-CSF-R) stimulated PI3K and activated Akt in macrophages and then activated Akt promoted collagen production in the BLM-induced mouse model. Reduced expression of Beclin-1, LC3A and LC3B and increased of p62 were also observed in a transgenic mouse model that expressed a constitutively-active form of Akt 100, indicating the pathogenic role of activated Akt in the regulation of autophagy and lung fibrosis. Forkhead box O3a (FoxO3a), a direct target of Akt, transcriptionally activated LC3-II by binding with the promoter region of LC3B. Of note, the expressions of FoxO3a and LC3-II were significantly reduced in fibroblasts from IPF patients, which subsequently reduced autophagy, promoted cell death and IPF progression 101. It has been reported that the expression of HDAC6 was observed to be increased in the BLM-treated mouse lungs and IPF lung tissues. As an HDAC6 inhibitor, tubastatin reversed the BLM-induced LC3B inhibition, S6k phosphorylation and HIF-1α expression, which ameliorated lung fibrosis by inhibiting the PI3K-Akt pathway in WT mice, but not in HDAC6 KO mice 102. Furthermore, recent studies also demonstrated a crucial role of the PDGFR-PI3K-Akt pathway in the activation of autophagy and regulation of myofibroblast differentiation. Genetic deletion of PARK2 inhibited mitophagy and enhanced myofibroblast differentiation and proliferation by activating the PDGFR-PI3K-Akt pathway in the BLM-induced mouse model 103. Similarly, pirfenidone, a PARK2 promoter, was observed to induce autophagy and inhibit myofibroblast differentiation by inducing PARK2 expression and reducing PDGFR-PI3K-Akt activation in BLM-treated WT mice, but not in PARK2 KO mice 104.

Aging is a significant risk factor contributing to higher mortality of IPF and dysregulated autophagy is believed to be involved in the progression of aging-related diseases. Compared with health control, LC3-II expression in fibroblasts from old IPF patients was downregulated due to increased S6K1 phosphorylation and hyperactivity of the mTOR pathway. Furthermore, modified starvation-induced autophagy, which further resulted in apoptosis resistance, was observed in the aging fibroblasts but not in the young fibroblasts. Altogether, mediated by mTOR activity, aging influenced the response of lung fibroblasts to autophagy and enhanced the vulnerability to lung fibrosis 105. More recent studies reported that sustained autophagy promoted fibroblast senescence and prevented the differentiation of fibroblasts to myofibroblasts through the regulation of mTOR complex 2 (mTORC2) 106. However, decreased degradation of p62 and impaired autophagy flux were also observed in senescent cells 107. In addition to aging and cell senescence, several obesity genes are also involved in the pathogenesis of IPF by regulating the mTOR pathway-related autophagy. Leptin promoted the EMT of lung epithelial cells and the TGF-β-induced production of Col-1 and α-SMA through the inhibition of autophagy. It was further demonstrated that leptin elevated the expression of p62, decreased autophagosome formation and inhibited lipidation of LC3-I to LC3-II by activating the PI3K/Akt/mTOR pathways 108. Intriguingly, LPS was also reported to inhibit autophagy and accelerate lung fibroblast proliferation by activating the PI3K-Akt-mTOR pathway, which was reversed by LY294002 and rapamycin 109. Wan and colleagues further found that LPS reduced autophagy and activated the PI3K/Akt/mTOR pathway *via* upregulating integrin β3 (Itgb3) and downregulating thymocyte differentiation antigen-1 (Thy-1) in the LPS-induced lung fibrotic mouse model and human lung fibroblast cells. Additionally, the effects of LPS on lung fibroblasts were respectively abolished by Thy-1 overexpression, a chemical inhibitor and specific shRNA of Itgb3 110.

Akt/mTOR-regulated autophagy may serve as a potential therapeutic option for the treatment of lung fibrosis. As mentioned above, ULK1 was regulated by the mTOR and AMPK pathways. A recent study has suggested that LKB1-AMPK-ULK1 axis-mediated autophagy plays a critical role in the BLM-induced lung fibrosis 111. Several studies have shown that several antioxidant agents and bioactive natural products, such as coenzyme Q 10 (CoQ10), celastrol and ligustrazin, promoted autophagy, reduced expression of TGF-β and Col-1 and protected against different chemical compounds-induced IPF through the downregulation of the PI3K/Akt/mTOR pathways in animal models 112-114. Du *et al.* further suggested that dioscin activated autophagy, reduced the secretion of inflammatory factors and subsequently alleviated the abnormal collagen accumulation. These effects of dioscin were abolished in alveolar macrophages when Atg5 was genetically depleted 115.

#### Interaction between activated autophagy and inflammation

Several studies suggest that inflammatory responses play essential roles in the interaction between IPF and autophagy. Angiotensin (Ang) II has been shown to aggravate lung fibrosis by activating the NLRP3 pathways. Meng *et al.* reported that activated autophagy attenuated the Ang II-induced activation of NLRP3 by eliminating ROS and alleviating mitochondrial dysfunction in the BLM-induced lung fibrosis, which was verified by rapamycin and 3-MA 116. In contrast to the promotive effects of Ang II on fibrosis, Ang-(1-7) attenuated smoking-induced lung fibrosis by activating autophagy flux and reducing NOX4-dependent ROS production in the passive smoking rat model and cigarette smoke extract-treated fibroblasts. These protective effects of Ang-(1-7) were abolished by autophagy inhibitors, 3-MA and bafilomycin A1 117. Toll-like receptor 4 (TLR4) is a critical player in the regulation of innate immunity. Recently, it has been revealed that activated TLR4 improved lung function and pulmonary fibrosis in the BLM-induced mouse model, which was abolished by TLR4 inhibitor TAK-242. Rapamycin treatment promoted the anti-fibrotic effects of TLR4 agonists, and in contrast, 3-MA treatment contributed to the effects of TLR4 antagonism 118. Furthermore, IL-17A was observed to promote the progression of lung fibrosis by regulating the interaction between inflammatory responses and autophagy. Physiologically, the phosphorylation of Ser70 of B-cell CLL/lymphoma 2 (Bcl-2) accelerated its dissociation from Beclin-1 so as to activate autophagy 119. IL17A inhibited the phosphorylation of Bcl-2 by activating PI3K-GSK-3β signaling cascade and thus blocked the activation of autophagy. Pharmacological inhibition of IL-17A suppressed lung fibrosis and accelerated collagen degradation by activating autophagy 120. On the other hand, as an anti-inflammatory cytokine, IL-37 was expressed in alveolar epithelial cells and alveolar macrophages in healthy controls but was significantly reduced in IPF patients. A recent study demonstrated that IL-37 decreased inflammation and inhibited collagen accumulation through the activation of autophagy in the BLM-induced mouse lung tissues 121.

## Cardiac fibrosis and autophagy

**Figure 5:**
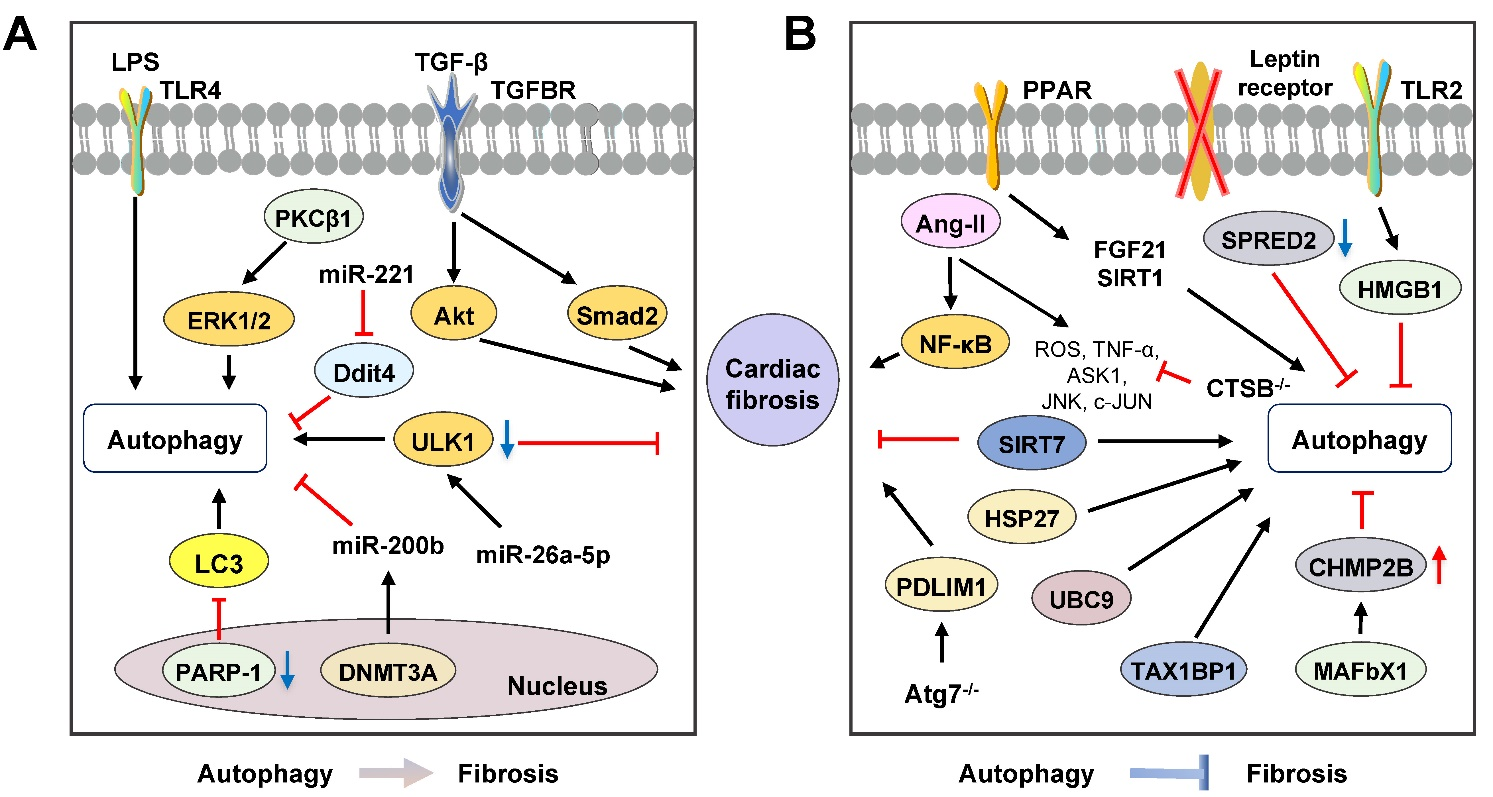
** Molecular links between autophagy and cardiac fibrosis.** Mounting evidence shows that autophagy plays contradictory roles in cardiac fibrosis. (A) The overexpression of several miRNAs, including miR-26a-5p and miR-221, or the downregulation of miR-200b suppress the activation of cardiac fibroblasts and autophagy flux. Furthermore, the activation of autophagy promotes cardiac fibrosis by regulating PARP-1, TGF-β/Smad and PKCβ1/ERK1/2 signaling pathways. (B) Accumulating evidence suggests that the activation of autophagy improves cardiac fibrosis illustrated by a variety of studies using specific-gene KO (CTSB, p8, Atg7 and SIRT7) mouse models. Panel B represents how autophagy-related signaling pathways, including SPRED2, PPAR, HMGB1, JNK/c-JUN, HSP27 and TAX1BP1/NF-κB pathways, and unfolded protein reactions contribute to cardiac fibrosis. CHMP2B: charged multivesicular body protein 2B; Ddit4: damage inducible transcript 4; DNMT3A: DNA methyltransferases 3A; HMGB1: high-mobility group box-1; PDLIM1: PDZ and LIM domain 1; SPRED2: sprouty-related protein with an EVH domain 2.

Cardiac fibrosis is a clinical manifestation of various cardiovascular diseases, including heart failure, cardiac arrhythmias and myocardial infarction. During the development of cardiac fibrosis, activation of cardiac fibroblasts and differentiation of fibroblast-to-myofibroblast result in the secretion of proinflammatory cytokines and fibrotic factors and ECM deposition 122. Increased α-SMA protein is assembled to stress fibers and remodel ECM, and consequently accelerates cardiac remodeling and heart failure. Notably, mounting evidence shows that autophagy plays a contradictory role in cardiac fibrosis** (Figure 5)**. Given that almost all heart diseases show fibrosis phenomenon and we mainly focus on the role of autophagy in fibroblast activation and cardiac fibrosis, articles regarding only fibrosis phenomenon involved in other heart diseases were excluded.

### Pro-fibrosis roles of autophagy in the heart

Emerging evidence suggests that the activation of autophagy is accompanied by cardiac fibrosis and targeting autophagy is a potential therapeutic option. A previous study reported that poly polymerase 1 (PARP-1), an extensive nuclear enzyme, accelerated the proliferation, migration and differentiation of cardiac fibroblasts induced by TGF-β. Genetic and pharmacological inhibition of PARP-1 decreased α-SMA expression and improved cardiac fibrosis through the downregulation of LC3 and Beclin-1 in the TGF-β-stimulated cardiac fibroblasts 122. Transverse aortic constriction (TAC) treatment was reported to increase the levels of Atg5, Atg16, LC3-II and Beclin-1, induce high levels of Ang II and heart fibrosis in the heart. Besides, aliskiren inhibited autophagy and thus attenuated heart fibrosis* via* downregulating the Ang II- protein kinase C β1 (PKCβ1)-ERK1/2 pathway in the TAC-induced mouse model, which was confirmed by 3-MA and PKCβ1 inhibitor LY333531 123. WD repeat and five domain containing 1 (WDFY1) is downstream of neuropilin-2, an important receptor in immune regulation. Cyclosporin A promoted myocardial fibrosis and increased the expressions of α-SMA, MMP-2/9, Col-1, TNF-α, LC3-II, Beclin-1 and ERK1/2 by activating autophagy, downregulating neuropilin-2 and upregulating WDFY1 in cardiac fibroblasts, which was abolished by 3-MA 124. Furthermore, a recent study demonstrated that LPS treatment upregulated the expression of autophagy markers and α-SMA in the cardiac fibroblasts after myocardial infarction. However, in TLR4 KO mice, the expressions of autophagy markers Atg5, Atg7, LC3-II, Beclin-1 were reduced and decreased autophagy was associated with the downregulation of fibrotic markers, such as α-SMA and vimentin 125.

#### Autophagy and microRNAs

Researchers have pointed out that miRNAs act as crucial regulators of cardiac homeostasis in heart diseases. In human cardiac fibroblasts, TGF-β treatment induced Col-1 and fibronectin synthesis by activating autophagy, which was facilitated by rapamycin and Atg5 overexpression and abolished by 3-MA and Beclin-1 knockdown 126. TGF-β receptor II (TGFBRII) was reported to transduce TGF-β1/2/3 signaling from cell membrane to cytoplasm. MiR-19a-3p suppressed ECM accumulation, cell invasion and phosphorylation of Smad2 and Akt through the deactivation of autophagy and downregulation of TGF-β RII signaling, which was confirmed by 3-MA and reversed by rapamycin 127. ULK1, a key player in the regulation of autophagy, is the target of miR-26a-5p. The overexpression of miR-26a-5p suppressed the expression of Col-1 and activation of LC3-I to LC3-II *via* downregulating the mRNA stability of ULK1 in rat primary cardiac fibroblasts 128. Coincidentally, recent studies confirmed that miR-221 reduced fibroblast activation and cardiac fibrosis by inhibiting damage inducible transcript 4 (Ddit4)-mediated autophagy in the hypoxia and reoxygenation-induced rat cardiac fibroblast activation, suggesting potential therapeutic effects of miR-221 on myocardial infarction-related fibrosis 129. Furthermore, miR-200b is another miRNA possessing anti-fibrotic effects by regulating autophagy. A previous study suggested that the expression of DNA methyltransferases 3A (DNMT3A), a protein of DNA methyltransferases family, was upregulated and miR-200b expression was decreased in rat cardiac fibroblast and fibrosis tissues. DNMT3A accelerated cardiac fibroblasts autophagy caused by rapamycin and cardiac fibrosis through the downregulation of miR-200b, which was abolished by miR-200b mimics and inhibition of DNMT3A in an abdominal aortic coarctation-induced cardiac fibrosis mouse model 130. Compared with fibrosis in other organs, although both the significance of miRNAs in the regulation of autophagy and the pathological role of excessive autophagy in the progression of cardiac fibrosis have been reported, the regulatory networks and interplay of miRNAs remain to be elucidated.

### Anti-fibrosis roles of autophagy in the heart

#### Autophagy dysfunction in myocardial hypertrophy-related cardiac fibrosis

Despite controversies, accumulating evidence suggests that the induction of autophagy may improve cardiac fibrosis as illustrated by a variety of pharmacological agonists/antagonists or specific-gene KO mouse models. Ang II contributes to cardiac fibrosis and graver cardiac remodeling, largely because of its promotive effects on myocardial hypertrophy. Ang II treatment induced the activation of rat cardiac fibroblasts by upregulating the expressions of Col-1 and fibronectin, increased ratios of LC3-II/LC3-I and LC3-II/p62 and the co-localization of LC3 puncta with vimentin in a mouse model. Pharmacological inhibition of autophagy by CQ not only enhanced the pro-fibrotic effects of Ang II but also augmented cardiac dysfunction, which was reversed by rapamycin treatment 131. Genetic abolishment of adiponectin (APN) increased the expressions of α-SMA, IL-1β, TNF-α and Col-1, promoted macrophage infiltration and decreased LC3-positive autophagosomes in the cardiac macrophages isolated from Ang II-induced mouse model. On the other hand, APN promoted macrophage autophagy and suppressed the Ang II-induced inflammatory responses and cardiac fibrosis through the activation of AMPK, which was abolished by AMPK inhibitor compound C 132. Recently a study showed that CTSB, a substrate of autophagy, was expressed in murine and human hearts, linking to myocardial infarction. Insufficient CTSB was shown to attenuate the cardiac remodeling and activation of TNF-α, ASK1, JNK and c-Jun signalings induced by Ang II in the CTSB KO mice 133. Autophagy-specific gene KO animals are also used to study the relationship between autophagy and myocardial hypertrophy-related cardiac fibrosis. Zhao *et al.* generated Atg5^+/-^ mouse model and confirmed that Ang II induced cardiac fibrosis and inflammation *via* increasing ROS production, macrophage accumulation, and the activation of NF-κB in macrophages was aggravated in Atg5^+/-^ mouse model 134. In recent years, it has become increasingly clear that MMPs play a crucial role in the pathogenesis of cardiovascular diseases. Transcriptional regulator p8 was observed to be increased in failing human hearts and stimulated MMP-9 induction. Georgescu *et al.* established p8 KO mouse model and confirmed that the deficiency of p8 attenuated cardiac fibrosis, decreased MMP-9 production and upregulated the expressions of MMP-2 and MMP-3 *via* accelerating expression of LC3-II and Atg12 in the TAC-stimulated mouse model 135. The involvement of TLR2 in Ang II-induced macrophage activation and cardiac fibrosis also has been noticed. It has been demonstrated that HMGB1 and TLR2 were co-localized in cardiac fibroblasts, accelerated the expressions of α-SMA and Col-1 and subsequently promoted cardiac fibrosis by inhibiting autophagy flux in the cardiac fibroblasts from the isoproterenol-induced myocardial hypertrophy-related cardiac fibrosis mouse model, which was prevented by silencing TLR2 136. Additionally, a novel cardiac hypertrophy mouse model was constructed by inducible endothelium-specific deleting of leptin receptors (End.LepR-KO). After applying transverse aortic constriction, activated autophagy, illustrated by elevated LC3-I/-II conversion and LC3-II positive endothelial cells in hearts, improved cardiac angiogenesis and suppressed cardiomyocyte hypertrophy were observed in End.LepR-KO mouse model. Interestingly, the inhibition of mTOR by rapamycin activated autophagy and the spontaneous cardiac hypertrophy in End.LepR-KO mice was improved 137.

#### Autophagy dysfunction contributes to fibrosis in proteotoxic cardiac diseases

The balance of protein synthesis and degradation is essential for cardiomyocyte homeostasis and is regulated by a large number of signaling cascades. Indeed, autophagy, UPR and ubiquitylation are key factors for this balance, whereas imbalance will inevitably result in cardiac dysfunction and fibrosis. Sprouty-related protein with an EVH domain 2 (SPRED2), an intracellular repressor of ERK-MAPK signaling, was expressed in the human heart and interacted with p62, a neighbor of BRCA1 (NBR1) and lysosomal cathepsin D thus participated in autophagolysosome formation. Insufficient SPRED2 reduced autophagy flux, dysregulated vesicle handing and promoted cardiac fibrosis by impairing the phagophore formation and inducing the accumulation of autophagosomal and lysosomal marker proteins in the SPRED2 KO hearts 138. Desmin-related cardiomyopathy (DRC), derived from interstitial fibrosis, is characterized by mutations in desmin or associated proteins and subsequent accumulation of cytotoxic misfolded proteins. Bhuiyan *et al.* established an enhanced Atg7 transgenic DRC mouse model and observed that the expression of Atg7 was increased in the DRC mouse model by crossing the two kinds of mice. They further confirmed that the Atg7-crossed DRC mouse model showed significantly reduced interstitial fibrosis and ventricular dysfunction accompanied by increased autophagy 139. Furthermore, ubiquitin/proteasome system (UPS) has been reported to mediate cardiomyocyte proteostasis. Ubiquitin-conjugating enzyme 9 (UBC9) is a small ubiquitin-like modifier (SUMO) E2 ligase and its expression improves SUMOylation-mediated and UPS-dependent cardiac protein quality control. Of note, the overexpression of UBC9 decreased cardiac fibrosis and improved cardiac function by increasing autophagy flux in the transgenic mice that overexpressed UBC9 in cardiomyocytes 140. Charged multivesicular body protein 2B (CHMP2B) is a protein participated in autophagosome formation. Mice lacking muscle-specific ubiquitin ligase atrogin-1 (also known as MAFbx1) failed to degrade CHMP2B, leading to autophagy impairment, UPR activation, cardiomyocyte apoptosis and subsequent cardiac fibrosis. The knockdown of CHMP2B restored autophagy flux and thus protected against atrogin-1 deficiency-induced cardiomyocyte death and interstitial fibrosis in the atrogin-1 KO mice 141.

#### Activation of autophagy improves cardiac fibrosis with different etiologies

Activation of autophagy is reported to attenuate myocardial infarction-related cardiac fibrosis. PDZ and LIM domain 1 (PDLIM1/CLP36), a negative cytoskeleton organization regulator, was accumulated in ischemia-reperfusion (IR) treated cardiomyocytes. Deficiency of Atg7 aggravated myofibrillar disarray, contractile dysfunction and severe cardiac fibrosis by inducing impaired degradation and accumulation of PDLIM1 in cardiomyocytes that specific disruption of Atg7 142. The activation of autophagy was found to reduce fibroblast differentiation and myocardial infarction-related cardiac fibrosis were observed in the SIRT7 KO mice and SIRT7 KO mouse-derived cardiac fibroblasts. Loss of Sirt7 downregulated the expressions of TGF-β RI, Smad2/3 and fibrosis-related genes, including α-SMA and fibronectin by increasing LC3 expression and activating autophagy flux, which was then abolished by CQ in SIRT7-siRNA treated cardiac fibroblasts 143.

Additionally, autophagy improves aging-related cardiac fibrosis. Heat shock protein 27 (HSP27) protected cardiac function from ischemia or chemical challenge and a mouse model with cardiac-specific expression of HSP27 was employed in the experiment of Lin *et al.* Studies with respect to HSP27 reported that it alleviated the aging-related interstitial fibrosis by increasing the expression of Atg13 and activating mitophagy in the HSP27 transgenic mouse model 144. Rho-associated coiled-coil-containing protein kinases (ROCKs), including ROCK1 and ROCK2, act as physiological regulators for vascular remodeling throughout aging. In ROCK1 and ROCK2 DKO mouse model, aging-related cardiac fibrosis was ameliorated accompanied with increased expression of LC3-II and suppressed Akt-mTOR pathway 145. Moreover, the development of cardiac aging was promoted by reducing the expressions of Atg5, Atg7, LC3-II and Beclin-1 and increasing p62 expression in Akt and AMPK DKO mice 146.

Furthermore, activation of autophagy attenuated fibrosis in diabetes-related cardiopathy. Decades of study have reported that numerous potential factors, including high circulating glucose, HFD, stress and inflammation in diabetes, contribute to susceptibility and progression of cardiopathy and cardiac fibrosis. Expression of tax1 binding protein 1 (TAX1BP1), a ubiquitin-binding protein that restricted NF-κB activation and inhibited inflammation, was decreased in diabetic mouse heart. The overexpression of TAX1BP1 ameliorated cardiac fibrosis and improved cardiac function by accelerating ratios of LC3-II/LC3-I and LC3-II/p62, which was reversed by 3-MA, CQ and bafilomycin A1 in the streptozotocin-induced diabetic cardiomyopathy mouse model 147. High glucose treatment, mimicking diabetes-related cardiopathy, was observed to increase the pro-fibrotic and inflammatory response, trigger oxidative stress and suppress autophagy in rat cardiomyocytes. Administration of fenofibrate (FF), a PPAR-α agonist, induced activation of autophagy and inhibited the fibrotic effects induced by high glucose through the upregulation of fibroblast growth factor 21 (FGF21)/SIRT1, which was reversed by 3-MA and SIRT1 inhibition 148.

## Kidney fibrosis and autophagy

**Figure 6:**
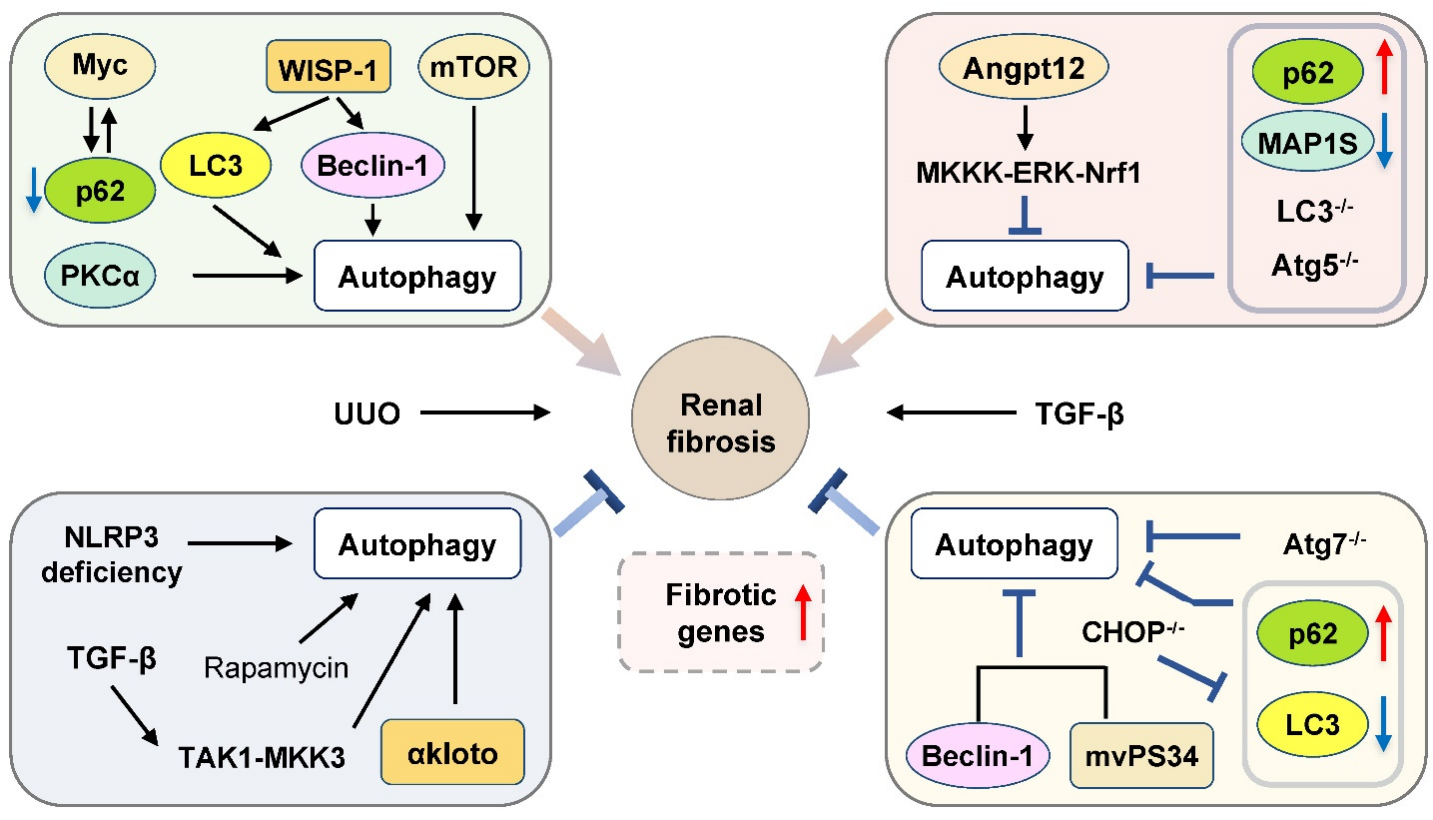
** Scheme diagram of autophagy in renal fibrosis.** This diagram represents contradictory roles of autophagy played in the pathogenesis of renal fibrosis depending on different experimental models, including pharmacological regulation and genetic modulation. Angpt12: angiopoietin-like protein 12; CHOP: C/EBP homologous protein; MAP1S: microtubule-associated protein 1S; mVps34: mammalian homologue of yeast vacuolar protein sorting defective 34; NLRP3: NOD-like receptors containing pyrin domain 3.

Kidney fibrosis is a histological hallmark and the final step of chronic kidney disease (CKD). Differentiation of resident fibroblast-to-myofibroblast, interstitial myofibroblast accumulation and ECM deposition are involved in kidney fibrosis 149, 150. Furthermore, renal tubular epithelial cells are perturbed in the pathological condition of acute kidney injury and contribute to kidney fibrosis by multiple mechanisms, including secreting inflammatory cytokines, growth and apoptosis factors and producing fibrogenic protein 150. Consistent with results in the liver, lung and heart, the function of autophagy in renal fibrosis remains ambiguous **(Figure 6)**.

### Pro-fibrosis roles of autophagy in the kidney

As many fibrogenic pathways are conserved across tissues, recent findings about autophagy flux in the liver, lung and heart could be further extended to studies of fibrosis in the kidney. Unilateral ureteral obstruction (UUO) is commonly used for the induction of renal fibrosis. Pharmacological inhibition of autophagy by CQ and 3-MA and autophagy disruption induced by genetic deletion of Atg7 in kidney proximal tubule cell prevented UUO-associated renal fibrosis. Consistent with these results, enhancement of autophagy by rapamycin promoted TGF-β-stimulated tubular cell death, interstitial inflammation and the production of α-SMA, fibronectin and vimentin in renal fibroblasts, which were reversed by CQ and 3-MA 149. Previous studies have also demonstrated that WNT family member 1 (WNT1)-induced signaling pathway protein-1 (WISP-1) was involved in fibrosis development in various organs. The overexpression of WISP-1 significantly increased the expression of LC3 and Beclin-1 and aggravated renal fibrosis both in the UUO-induced mouse model and TGF-β-stimulated tubular epithelial cells. Anti-WISP-1 antibody treatment suppressed the activation of autophagy and improved renal injury in the UUO-stimulated rat model 151. mTORC2 and PKCα, one of the major sub-pathways of mTORC2, play a crucial role in the fibroblast activation and renal fibrosis. A recent study demonstrated that activated PKCα accelerated renal fibrosis development by increasing the expression of LAMP-2, autophagosome-lysosome fusion and autophagy flux, which was abolished by PKCα inhibitor Go6976 and PKCα-siRNA in the TGF-β-induced fibroblasts and UUO-stimulated mouse model 150. Myc gene was activated and interacted with p62 to induce functional metabolic derangement and following activation of kidney stromal cells after UUO-induced renal injury. IL-1β treatment stimulated autophagy flux, as indicated by the increased ratios of LC3-II/LC3-I and LC3-II/p62, resulted in the degradation of p62 and accumulation of Myc and subsequently induced tubular fibrosis, which was reversed by the inhibition of IL-1R signal transducer kinase 4 (IRAK4) or Myc 152. Furthermore, in C/EBP homologous protein (CHOP) KO mice, Noh *et al.* demonstrated that the expression of Beclin-1, LC3-I, LC3-II and p62, and renal cell apoptosis caused by UUO treatment were reduced and renal fibrosis was alleviated 153.

Autophagy is also implicated in the regulation of IR injury-related kidney fibrosis. Baisantry *et al.* developed a transgenic mouse model with specific deletion of Atg5 in the proximal tubular S3 segments and demonstrated that these mice were protected against IR injury-induced tubular senescence, cell death, and interstitial fibrosis. Interestingly, due to autophagy-mediated the malfunction of fibrosis-related tissue repair, the long-term outcome of these mice after IR injury was worse than WT control mice, as indicated by severe inflammation and development of senescent phenotype 154. Previous studies suggested that proximal epithelial cell (PTC) arrested at G2/M phase is critical for the secretion of fibrotic factors during kidney injury. Recently, Canaud *et al.* observed that autophagy-dependent formation of mTOR-autophagy spatial coupling compartments (TASCCs) and following mTORC1 activation contributed to the increased secretion of inflammatory and fibrotic proteins in PTC in both IR-induced kidney injury and human chronic kidney diseases. Genetic depletion of Rapter, a key component in the formation of TASCCs, attenuated kidney fibrosis 155.

The promotive effects of autophagy on kidney injury-related fibrosis in other experimental models have also been investigated. The mammalian homologue of yeast vacuolar protein sorting defective 34 (mVps34), a class III PI3K, was reported to combine with Beclin-1 and participate in the regulation of autophagy. It has been shown that specific KO of mVps34 in the podocytes resulted in complemental increased autophagosome and lysosomes and these mice developed obvious podocyte vacuolization, glomerulosclerosis and interstitial fibrosis by 6 weeks of age and died before 9 weeks of age 156. In addition, sodium hydrosulfide protected against chronic renal injury by ameliorating oxidative status, reducing the mRNA levels of LC3-II and Beclin-1 and apoptosis in a 5/6 nephrectomy rat model 157.

### Anti-fibrosis roles of autophagy in the kidney

Pharmacological activation of autophagy by rapamycin attenuated Col-1 accumulation and kidney fibrosis in the UUO-induced mouse model and Ang II-treated epithelial cells. Additionally, several whole body or cell-specific autophagy gene KO animals were developed to identify the role of autophagy in renal fibrosis. Genetic inhibition of autophagy by proximal tubular epithelial cell-specific Atg5 KO mice were highly susceptible to UUO-induced kidney fibrosis, concomitant with cellular senescence including mitochondrial dysfunction, mitochondrial DNA abnormalities, nuclear DNA damage, and cell cycle arrest at the G2/M phase 158, 159. Concurrently, Peng *et al.* applied the same cell-specific Atg5 KO mice and confirmed that the deficiency of Atg5 markedly impaired autophagy, produced more cytokines and accelerated macrophage and lymphocyte infiltration by inducing the nuclear translocation of p65 and transcriptional activity of NF-κB in the obstructed kidneys after UUO treatment 160. Furthermore, endothelial-specific Atg5 KO mice displayed IL-6-dependent endothelial mesenchymal transition and severe renal fibrosis. Neutralization of IL-6 by monoclonal antibody promoted autophagy and alleviated fibrosis, which was reversed by 3-MA 161. In addition to specific Atg5 KO mice, Nam *et al.* further constructed a tubular epithelial cell-specific Atg7 deletion mouse model and demonstrated that impaired autophagy accelerated renal tubulointerstitial fibrosis and distal tubules EMT-like phenotype change *via* increasing the expression of TGF-β, IL-1β and NLRP3 in the kidney after UUO treatment 162. Mitochondrial dysfunction and ER stress were reported to be involved in the progression of focal segmental glomerular sclerosis (FSGS), a heterogeneous kidney disease. In the renal epithelium cell-specific Atg5 or Atg7 KO mice, mitochondrial dysfunction was observed in association with ER stress and oxidative stress and the incidence of spontaneous FSGS was elevated 163.

Similar to the findings in the liver, a recent study further identified that MAP1S-related autophagy is involved in the regulation of renal fibrosis. The MAP1S levels were significantly reduced in the renal fibrotic tissues from renal failure patients and the autophagy flux was impaired. As expected, the depletion of MAP1S disrupted autophagy-dependent clearance of fibronectin and induced fibronectin accumulation during renal fibrosis 164. In renal tubular cells, hypoxia induced mitochondrial localization of NLRP3 and its binding to mitochondrial antiviral signal protein (MAVS), which facilitated mitochondrial ROS production and cell apoptosis. The deletion of NLRP3 suppressed the UUO-induced renal fibrosis and decreased the renal tubular cell apoptosis by increasing the autophagy-dependent ROS clearance 165.

Notably, recent studies have yielded many important advances in the understanding of the role of age-dependent autophagy in cellular senescence and fibrosis 166, 167. A previous study suggested that although basal autophagy activity was higher in the aged kidney, autophagy flux and protein turnover were inhibited with aging, as illustrated by the accumulation of p62 and ubiquitin-positive proteins. Accumulation of misfolded proteins further contributed to cell senescence and potentiated renal fibrosis 158. α-Klotho, a type 1 transmembrane protein, is originally identified as an aging suppressor and can be released into circulation and exert multiple effects on target organs.α-Klotho expression deficiency was shown to be responsible for endothelial dysfunction, microangiopathy and fibrosis 168. Similarly, a recent article showed that αKlotho activation attenuated renal fibrosis, protected renal cells from oxidative stress and suppressed the progression of AKI to CKD through the upregulation of autophagy flux, which was reversed in α-Klotho deficiency mouse model 169. Autophagy was also implicated in the fibrogenesis in diabetic nephropathy. Angiopoietin-like protein 12 (Angpt12), a member of the Angptl family, has a critical function in physiological tissue remodeling. Activated Angptl2 downregulated autophagy, increased pro-inflammatory factors, accelerated renal fibrosis by activating the MKKK-ERK-Nrf1 pathway, which was completely reversed by Angptl2 knockdown in a diabetic rat model 170. Additionally, calorie restriction decreased the accumulation of oxidized GSH, ameliorated tubulointerstitial macrophage infiltration and renal fibrosis by promoting autophagy in *ob/ob* diabetic mice 171.

#### Autophagy and the TGF-β-related pathways

Emerging evidence suggests that TGF-β treatment not only stimulates collagen deposition but also induces autophagy in the primary mouse and human tubular epithelial cells. Ding *et al*. further constructed LC3^-/-^ or Beclin-1^+/-^ mouse model and identified that the disruption of autophagy promoted collagen accumulation and the expression of TGF-β in the UUO mouse model 172. Previous studies also demonstrated a crucial role of autophagy played in the transcriptional regulation of collagen expression. TGF-β-activated kinase 1 (TAK1), a serine/threonine kinase, belongs to the MAPK kinase kinase (MKKK) family. In addition to the Smad signaling pathways, TGF-β phosphorylates and activates TAK1 in a Smad-independent manner and subsequently triggers downstream signaling cascades. Recently, researchers reported dual roles of TGF-β that TGF-β not only induced Col-1 accumulation but also promoted autophagy and Col-I degradation by inducing the TAK1-MAPK kinase 3 (MKK3) pathway in primary mouse mesangial cells, which was confirmed in the MKK KO mice. Dysfunction of autophagy induced by heterozygous deletion of Beclin-1 increased the protein levels and accumulation of Col-1 in primary mouse mesangial cells, indicating that the intracellular degradation of Col-I and insoluble pro-collagen I was at least partly dependent on autophagy 173.

## Autophagy and cystic fibrosis

Cystic fibrosis, as a most frequent lethal genetic disorder, is induced by the mutations in the CFTR gene and affects multiple organs, including the lung, liver, kidney, pancreas and intestine 174. The most common observed mutation of CFTR is a phenylalanine deletion at position 508, which leads to protein misfolding, incomplete glycosylation, impaired cell-surface trafficking and eventually abolished the function of CFTR as a plasma membrane located ion channel. Although rare, mutation-mediated CFTR dysfunction is implicated in the dysregulation of glucose metabolism, impairment of insulin release, complications in lung and airway and subsequent multiorgan failure, which contribute to the high mortality of cystic fibrosis 175.

### Pro-fibrosis roles of autophagy in cystic fibrosis

Cystic lung fibrotic disease is considered as a hypersecretory inflammatory disease characterized by airway obstruction and treatments aiming to control chronic inflammation and decrease excess mucus are effective strategies to reverse cystic fibrosis. In a recent report, IL-13 aggravated cystic fibrosis-induced airway inflammatory by accelerating the formation of airway goblet cells that secreted excess mucus. Notably, the inhibition of autophagy by 3-MA and bafilomycin A1 attenuated mucus secretion in the IL-13-induced mouse model, which was further confirmed by Atg5 or Atg16 depleting 175.

### Anti-fibrosis roles of autophagy in cystic fibrosis

It has been well-established that the failure of autophagy is closely associated with the pathogenesis of cystic fibrosis. It has been reported that autophagy was impaired in cystic fibrosis patients and cystic fibrosis mice, as illustrated by downregulated intracellular expression of essential autophagy molecules including LC3-II, Beclin-1 and Atg5 176. On the other hand, defective CFTR function further impaired autophagy in cystic fibrosis patients. In the airway epithelial cells of patients with cystic fibrosis, dysfunction of mutated CFTR stimulated Beclin-1 reduction and p62 accumulation *via* upregulating ROS and tissue transglutaminase (TG2) 177.

A growing number of studies suggested that restoring autophagy presented beneficial effects against cystic fibrosis development. Under cellular ER stress and an ER-to-Golgi transport blockade, immature mutated CFTR was transported to cytomembrane from ER through an unconventional route bypassed Golgi, which partially restored the function of mutated CFTR as an ion channel. Interestingly, this unconventional trafficking is dependent on the activation of autophagy. Autophagy inhibition mediated by 3-MA, wortmannin, and genetic deletion of Atg5 and Atg7 significantly abolished the unconventional secretion of CFTR 178. The binding of Bcl2-associated athanogene (BAG) 3 with HSP70 is critical for the UPS- and autophagy-dependent misfolded proteins degradation. The knockdown of BAG3 by specific siRNA not only restored autophagy in cystic fibrosis but also functionally corrected mutated CFTR by enhancing its stability and facilitating its trafficking. Compromising autophagy by Atg7 siRNA inhibited the protective effects of BAG3 silencing 179. It has been reported that Atg7 and Atg16 were targets of miR-17-92 and downregulation of miR-17-92 improved CFTR channel function and reversed the compromised ability of transport halide through the activation of autophagy in macrophages 174.

Cystic fibrosis patients are more susceptible to infection induced by bacteria, including *P seudomonas aeruginosa* and *B.cenocepacia*. Emerging evidence suggested that the dysfunction of mutated CFTR and defective autophagy synergistically compromised internalization and clearance of bacteria by macrophages and epithelial cells. Ferrari *et al.* demonstrated that proteostasis regulator cysteamine partly rescued the function of mutated CFTR, enhanced macrophages-mediated bacteria clearance and ameliorated inflammation through the upregulation of Beclin-1 and activation of autophagy 180. Furthermore, pyocyanin is a well-described *P. aeruginosa* virulence factor playing an important role in the airway infection and initial inflammation in cystic fibrosis patients and significantly induces autophagy through eIF2α-ATF4 pathway. Interestingly, inhibition of autophagy by CQ significantly increased bacteria colonization, and the mortality rate of *P. aeruginosa* infection, suggesting that activation of autophagy is an adaptive and protective mechanism 181. Furthermore, the activation of autophagy induced by epigallocatechin-3-gallate restricted the *B.cenocepacia* replication and subsequently ameliorated cystic fibrosis by suppressing the methylation of Atg12 promoter and increasing Atg12 expression 176.

## Conclusion and future prospection

**Figure 7:**
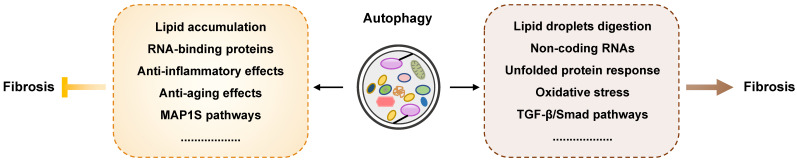
** Generalized relations between autophagy and fibrosis.** Fibrosis: liver fibrosis, lung fibrosis, cardiac fibrosis, renal fibrosis and cystic fibrosis.

Fibrosis is a chronic disease characterized by an excessive accumulation of ECM and is a hallmark feature of pathologic remodeling in numerous tissues. Although the specific symptoms of fibrosis are diverse in each organ, the pathogenesis across organs shares several common mechanisms, including but not limited to excessive activation of fibroblasts, conversion of fibroblasts to myofibroblasts and the proliferation of ECM-producing myofibroblasts. As a complex and essential catabolic process, autophagy occurs in most mammalian cells and is considered to be a crucial regulator of multiorgan fibrotic diseases. However, based on all the evidence summarized above, it is not difficult to see the controversial role of autophagy in the regulation and development of fibrotic diseases (**Figure 7**). Some studies have shown that autophagy participates in lipid droplet digestion and energy supply for fibroblast activation and can aggravate fibrosis, whereas other studies have revealed that activated autophagy may attenuate inflammatory responses and ameliorate fibrosis in multiple organs. Considering the contradictory role of autophagy in the pathogenesis of almost all types of fibrotic diseases, the identification of downstream specific targets or signaling pathways of autophagy remains crucial for the treatment of fibrosis.

Fibrotic diseases are commonly progressive, and their pathological manifestations at different stages of fibrosis vary dramatically. LDs are stored in quiescent fibroblasts at an early step in fibrosis but not in activated myofibroblasts. Concurrently, the breakdown of intracellular lipids and the satisfaction of energy requirements are indispensable to establish an activated fibroblast phenotype. Thus, functions and susceptibility to activation of autophagy may be different in the diverse stages of fibrosis. Moreover, given that aging may affect the alteration of autophagy activity and concurrently increase the risk of fibrosis, comparing and analyzing experimental results between adult and aged mice is unpersuasive and less rigorous. In addition, numerous studies have demonstrated that multiple signaling pathways are involved in the activation and development of fibrosis. Considering the simultaneous effects of these diverse signaling pathways on the regulation of autophagy, it is essential to distinguish the autophagy-mediated effects from secondary compensating actions. Taking liver fibrosis as an example, although lncRNA H19 activation accelerated liver fibrosis and concurrently triggered autophagy by activating the PI3K/Akt/mTOR pathway, it is inappropriate to tell whether activated autophagy in this mouse model is the pathogenic mechanism or a compensatory response against fibrosis. Similarly, due to the specific anti-fibrotic effects of TGF-β inhibition itself, it is inaccurate and subjective to conclude that inhibition of autophagy can ameliorate liver fibrosis in studies that simultaneously blocked autophagy and TGF-β pathways. In addition, the exact role of autophagy in fibrogenesis is dependent on specific cell types and different stimuli. Gao *et al.* indicated that autophagy was inhibited in PDGF- but not TGF-β-stimulated HSCs, leading to enhanced release of fibrogenic EVs 53. Consistent with these results, Allaire *et al.* confirmed that the role of autophagy in fibrosis is more complicated than originally thought and probably depends on specific cell types 5. Further identification and evaluation of the function of autophagy in different cell types and specific stimuli will improve the understanding of the role of autophagy in fibrosis.

Mounting evidence showed that autophagy flux was considered to be a dynamic process of autophagy, involving the formation and secretion of autophagosomes, the phagocytosis of cargo and the formation and degradation of autolysosomes. Compared with the expression of autophagy markers, the change in autophagy flux, as assessed by ratios of LC3-II/LC3-I and LC3-II/p62, more adequately represent the activity of autophagy. Interestingly, several studies demonstrated that although LC3-II accumulation was activated, blocking autophagy flux and inhibited autophagy activity were observed* in vivo* and* in vitro* 182, 183, suggesting the need to measure autophagy flux in future studies. As reviewed above, the suppressive effects of increased autophagy flux on fibrosis in multiple organs, including the liver, lung, heart and kidney have been consistently observed. However, the contrasting results of experiments have described the promoting effects of increased autophagy on liver and kidney fibrosis, where only the expression of autophagy markers was examined and autophagy flux was rarely assessed, indicating that these studies were not comprehensive and objective.

Certainly, experimental evidence obtained from the specific-autophagy genes, like Atg genes, KO mice are necessary and convincing to elucidate the role of autophagy in the fibrosis process. Considering that embryonic or neonatal lethality has been detected in conventional Atg gene KO mice, conditional tissue/cell-specific or whole-body gene KO was performed in adult mice 184. Interestingly, due to the lower mortality of Atg5 and Atg7 KO mice compared with that of mice with other gene deletions, previous studies used Atg KO mice, mostly targeting Atg5 and Atg7, but rarely used LC3 and Atg4b KO mice. Most of these studies showed that impaired autophagy promoted inflammation, cell apoptosis, ROS production and subsequently aggravated multiorgan fibrosis, while a few studies reported that autophagy inhibition ameliorated liver fibrosis and cell senescence in the kidney. It is noteworthy that Atg gene KO, regardless of whether they are global or tissue/cell-specific, induce complete and irreversible abrogation of autophagy, which does not generally exhibit the etiology of human diseases. Thus, these issues bring substantial obstacles to translate observations from Atg KO mouse experiments to clinical therapies of fibrotic diseases.

Collectively, emerging evidence has confirmed and made substantial strides in promoting a better understanding of the role of autophagy in multiorgan fibrosis, including liver, lung, cardiac, renal and cystic fibrosis. Although the anti-fibrotic function of autophagy remains a controversial topic, it is indisputable that autophagy has increasingly become a promising therapeutic target of fibrotic diseases. Therefore, characterizing and emphasizing the function of autophagy in fibrosis not only provides a novel framework for the appreciation and exploration of pathogenesis in fibrosis but also is urgently required for the discovery of potential clinical treatments of multiorgan fibrosis.

## Abbreviation

AMPK: AMP-activated protein kinase
Ang II: angiotensin II
Akt: protein kinase B
Atg: autophagy-related gene
BDL: bile duct ligation
BLM: bleomycin
CCl_4_: carbon tetrachloride
CQ: chloroquine
Col-1: collagen type I
CFTR: cystic fibrosis transmembrane conductance regulator
ECM: extracellular matrix
EMT: epithelial-mesenchymal transition
ERK1/2: extracellular signal-regulated kinase1/2
HSC: hepatic stellate cell
IPF: idiopathic pulmonary fibrosis
LC3: light chain 3
LD: lipid droplet
LPS: lipopolysaccharide
MAPK: mitogen-activated protein kinase
mTOR: mammalian target of rapamycin
PI3K: phosphatidylinositide3-kinase
TAK1: TGF-β-activated kinase 1
TGF-β: Transforming growth factor-beta
TLR: toll-like receptor
UUO: unilateral ureteral obstruction
